# Hepatocyte HSPA12A inhibits macrophage chemotaxis and activation to attenuate liver ischemia/reperfusion injury via suppressing glycolysis-mediated HMGB1 lactylation and secretion of hepatocytes

**DOI:** 10.7150/thno.82607

**Published:** 2023-07-03

**Authors:** Shuya Du, Xiaojin Zhang, Yunxiao Jia, Peipei Peng, Qiuyue Kong, Surong Jiang, Yuehua Li, Chuanfu Li, Zhengnian Ding, Li Liu

**Affiliations:** 1 Department of Geriatrics, Jiangsu Provincial Key Laboratory of Geriatrics, the First Affiliated Hospital of Nanjing Medical University, Nanjing 210029, China.; 2 Department of Anesthesiology, First Affiliated Hospital of Nanjing Medical University, Nanjing 210029, China.; 3 Key Laboratory of Targeted Intervention of Cardiovascular Disease, Collaborative Innovation Center for Cardiovascular Disease Translational Medicine, Nanjing Medical University, Nanjing 210029, China.; 4 Departments of Surgery, East Tennessee State University, Johnson City, TN 37614, USA.

**Keywords:** Hepatocyte, Macrophage, Chemotaxis, Liver ischemia/reperfusion (LI/R), Lactylation, High-mobility group box 1(HMGB1), Heat shock protein A12A (HSPA12A)

## Abstract

**Rationale:** Liver ischemia-reperfusion (LI/R) injury is characterized by two interconnected phases: local ischemia that causes hepatic cell damage to release damage-associated molecular pattern (DAMPs), and DAMPs that recruit immune cells to elicit inflammatory cascade for further injury of hepatocytes. High-mobility group box 1 (HMGB1) is a representative DAMP. Studies in macrophages demonstrated that HMGB1 is secreted after lactylation during sepsis. However, whether lactylation mediates HMGB1 secretion from hepatocytes after LI/R is known. Heat shock protein A12A (HSPA12A) is an atypical member of HSP70 family.

**Methods:** Gene expression was examined by microarray analysis and immunoblotting. The hepatic injury was analyzed using released ALT and AST activities assays. Hepatic macrophage chemotaxis was evaluated by Transwell chemotaxis assays. Inflammatory mediators were evaluated by immunoblotting. HMGB1 secretion was examined in exosomes or serum. HMGB1 lactylation was determined using immunoprecipitation and immunoblotting.

**Results:** Here, we report that LI/R decreased HSPA12A expression in hepatocytes, while hepatocyte-specific HSPA12A overexpression attenuated LI/R-induced hepatic dysfunction and mortality of mice. We also noticed that hepatocyte HSPA12A overexpression suppressed macrophage chemotaxis to LI/R-exposed livers *in vivo* and to hypoxia/reoxygenation (H/R)-exposed hepatocytes *in vitro*. The LI/R-increased serum HMGB1 levels of mice and the H/R-increased HMGB1 lactylation and secretion levels of hepatocytes were also inhibited by hepatocyte HSPA12A overexpression. By contrast, HSPA12A knockout in hepatocytes promoted not only H/R-induced HMGB1 lactylation and secretion of hepatocytes but also the effects of H/R-hepatocytes on macrophage chemotaxis and inflammatory activation, while all these deleterious effects of HSPA12A knockout were reversed following hepatocyte HMGB1 knockdown. Further molecular analyses showed that HSPA12A overexpression reduced glycolysis-generated lactate, thus decreasing HMGB1 lactylation and secretion from hepatocytes, thereby inhibiting not only macrophage chemotaxis but also the subsequent inflammatory cascade, which ultimately protecting against LI/R injury.

**Conclusion:** Taken together, these findings suggest that hepatocyte HSPA12A is a novel regulator that protects livers from LI/R injury by suppressing glycolysis-mediated HMGB1 lactylation and secretion from hepatocytes to inhibit macrophage chemotaxis and inflammatory activation. Therefore, targeting hepatocyte HSPA12A may have therapeutic potential in the management of LI/R injury in patients.

## Introduction

Liver ischemia/reperfusion (LI/R) injury is a major complication of hepatectomy and liver transplantation, as well as of abdominal trauma, hemorrhagic shock, and myocardial ischemia 1. LI/R injury can cause not only acute hepatic dysfunction but also chronic disorders such as hepatic fibrosis/cirrhosis 2, 3. With regard to liver transplantation, LI/R injury is an important risk factor for graft rejection 4. Therefore, mitigating LI/R injury would improve patient prognosis and preserve the donor pool available for life-saving liver transplantation. Despite its remarkable clinical importance, LI/R injury still lacks effective therapeutic interventions.

LI/R injury is a dynamic process that involves two interconnected phases: local ischemic insult and inflammation-mediated reperfusion injury 5. In the initial phase of LI/R, ischemic insult damages hepatic cells. These damaged cells then release DAMPs (damage-associated molecular patterns) to recruit immune cells from the circulation after reperfusion, and this elicits a sterile inflammatory cascade that aggravates hepatocellular damage and causes reperfusion injury 4, 5. Macrophages are a major class of immune cells, and their recruitment and activation are central to this inflammatory cytotoxic cycle that causes widespread cell death in LI/R-exposed livers 4. Therefore, suppressing DMAPs release to disrupt “hepatocyte damage-macrophage recruitment/activation-hepatocyte damage” cytotoxic cycle presents a promising strategy for limiting LI/R injury.

High-mobility group box 1 (HMGB1) is a representative DAMP. Under normal conditions, HMGB1 locates to the cell nucleus and functions as a DNA-scaffolding protein 6, 7. However, nuclear HMGB1 can be secreted into the extracellular space, and extracellular HMGB1 is a necessary and sufficient mediator to trigger inflammation through recruitment and activation of macrophages and other immune cells 8-13. Of note, HMGB1 can also be secreted by hepatocytes after LI/R, and HMGB1 inhibition provides significant protection against LI/R injury 14, 15. However, it is not clearly understood regarding the regulation of HMGB1 secretion from hepatocytes under LI/R condition. Intriguingly, we have demonstrated that in macrophages during sepsis, HMGB1 is secreted in exosomes after its lactylation 10. Lactylation is a newly identified, p300-mediated post-translational modification that adds lactyl groups to proteins using glycolysis-derived lactate 10, 16. However, in hepatocytes of LI/R-exposed livers, whether lactylation mediates HMGB1 secretion and how HMGB1 lactylation might be regulated are completely unknown.

Heat shock protein A12A (HSPA12A) is a distant member of the HSP70 family 17. We demonstrated that HSPA12A encodes a prosurvival pathway against endotoxemic liver injury 18. Particularly, in cancer cells, we found that glycolytic activity is regulated by HSPA12A 19, 20. Glycolysis is a metabolic process that converting glucose to pyruvate which can subsequently convert to lactate. Considering that lactate increased HMGB1 lactylation and secretion of macrophage during sepsis 10, it is possible, therefore, that HSPA12A modulates glycolysis-derived lactate to affect HMGB1 lactylation and secretion from hepatocytes, by which changes macrophage chemotaxis and activation, and finally affects LI/R injury.

To test this hypothesis, we performed experiments using primary hepatocytes and knockin mice (h-Ki) with hepatocyte-specific overexpression of HSPA12A. We found that the increased liver macrophage chemotaxis was accompanied by decreased hepatocyte HSPA12A expression following LI/R, whereas h-Ki mice displayed attenuation of the LI/R-induced macrophage chemotaxis, hepatic dysfunction, and mouse mortality. Further gain- and loss-of-function studies revealed that under a LI/R-like condition, hepatocyte HSPA12A was essential for disrupting the inflammatory cytotoxic cycle between hepatocyte damage and macrophage chemotaxis/activation, and this hepatoprotective action of HSPA12A was mediated through inhibiting glycolysis-generated lactate to decrease HMGB1 lactylation and exosomal secretion of hepatocytes. Therefore, strategies that increasing hepatocyte HSPA12A has therapeutic potential in the clinical management of LI/R injury.

## Results

### Increased macrophage chemotaxis is accompanied by decreased hepatocyte HSPA12A expression following LI/R

To establish optimal experimental conditions for the LI/R study, mice were subjected to liver ischemia for 1 h followed by reperfusion for 3 and 6 h. Serum aminotransferase (AST) and alanine aminotransferase (ALT) activities increased sharply following 3 h of reperfusion and further slightly increased following 6 h of reperfusion (**Figure S1A**). To simulate LI/R *in vitro*, primary hepatocytes were exposed to hypoxia for 6 h followed by reoxygenation for 3, 6, 9, and 12 h. Again, extracellular ALT and AST activities increased greatly following 3 h of reoxygenation and remained at the same levels for up to 12 h of reoxygenation (**Figure S1B**). Thus, LI/R for 1 h/3 h was selected for experiments in mice *in vivo*, and hypoxia/reoxygenation (H/R) for 3 h/3 h was used for primary hepatocytes to simulate LI/R *in vitro*.

**Figure 1:**
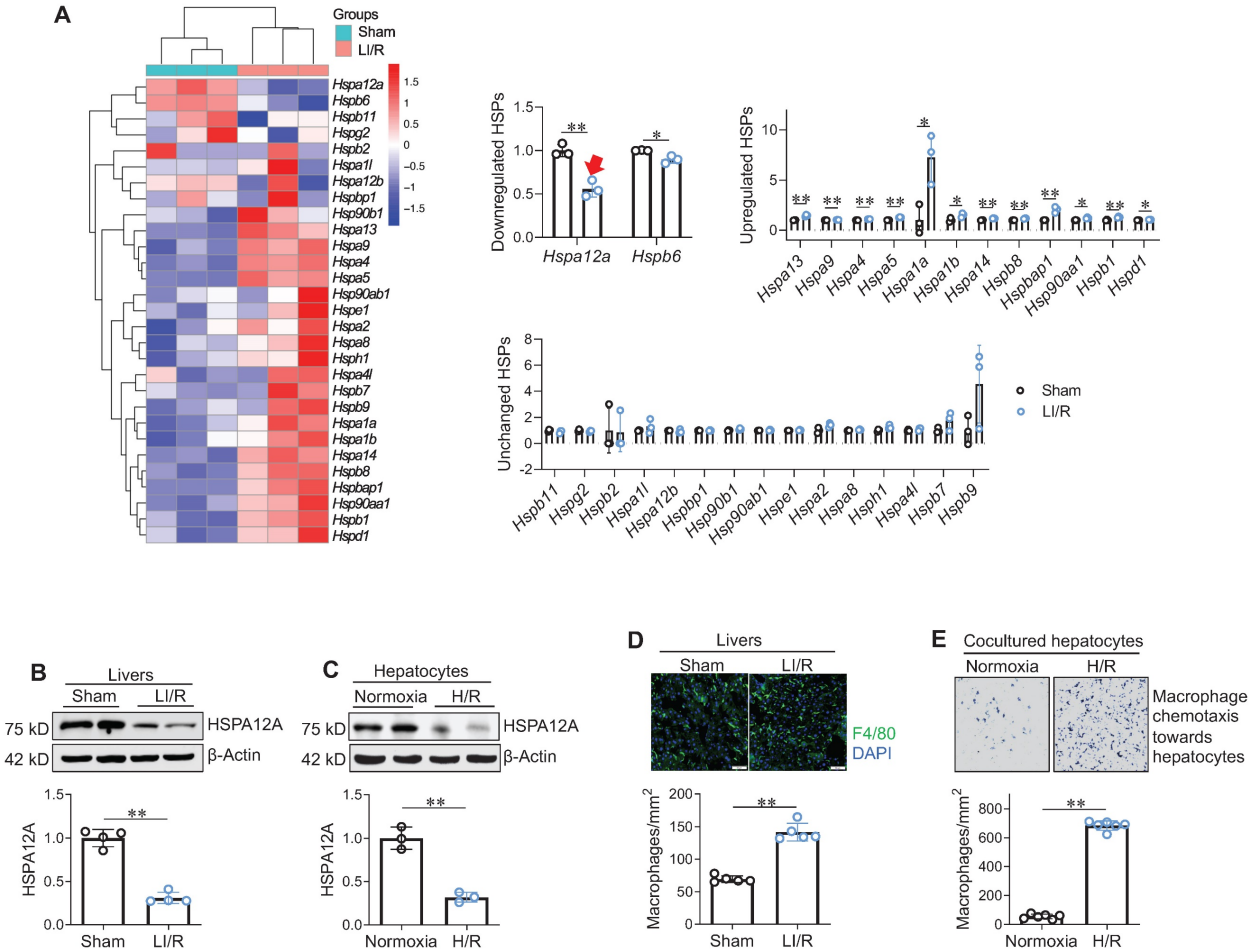
** Increased macrophage chemotaxis was accompanied with decreased hepatocyte HSPA12A expression following LI/R. A. *Hsp* mRNA data set from microarray analysis.** Livers from sham and LI/R mice were applied to microarray analysis. *Hsp* mRNA data set from microarray was heat mapped and statistically analyzed. The red arrow indicates the decrease of *hspa12a* mRNA expression. n = 3/group. **B. HSPA12A protein expression in livers.** HSPA12A protein expression was examined in mouse livers after sham or LI/R. n = 4/group. **C. HSPA12A protein expression in H/R hepatocytes.** HSPA12A protein expression was examined in primary hepatocytes after normoxia or H/R. n = 3/group. **D. Macrophage recruitment in livers.** Immunostaining for macrophage marker F4/80 was performed on cryosections that prepared from mouse livers after LI/R. DAPI was used to stain nuclei. Scale bar = 50 μm. n = 5 /group. **E. Macrophage chemotaxis towards hepatocytes.** H/R- or normoxia-exposed primary hepatocytes were cocultured with primary macrophages in Transwell plates for 24 h. The macrophage chemotaxis towards hepatocytes was stained with crystal violet. n = 6/group. Data are mean ± SD, ** *P* < 0.01 and * *P* < 0.05 by Student's two-tailed unpaired *t* test.

Next, effect of LI/R on hepatic HSP expression profile was examined using microarray analysis: among the 29 *Hsps* measured, mRNA expression was downregulated for 2 *Hsps*, upregulated for 12 *Hsps*, and unchanged for 15 *Hsps* following LI/R (**Figure 1A**). Notably, *Hspa12a* mRNA levels showed a greater magnitude of decrease than the other downregulated *Hsp* (*Hspb6*) after LI/R. This observation was confirmed by immunoblotting: protein levels of HSPA12A were lower in mouse LI/R livers and H/R hepatocytes than their controls (**Figure 1B-C**). Meanwhile, macrophage recruitment to mouse LI/R livers and to H/R hepatocytes was increased than their controls (**Figure 1D-E**). Therefore, downregulated hepatocyte HSPA12A may be associated with LI/R-induced macrophage chemotaxis and hepatic injury.

### Hepatocyte HSPA12A overexpression improves mouse survival and attenuates hepatic injury after LI/R

To determine the direct role of hepatocyte HSPA12A in LI/R injury, we created knockin mice (h-Ki) with hepatocyte-specific overexpression of HSPA12A (**Figure S2A-C**). HSPA12A was overexpressed specifically in livers of h-Ki mice (**Figure S3A**). In h-Ki livers, HSPA12A was overexpressed in hepatocytes but not in macrophages (**Figure S3B**). Compared with wild type (WT) littermates, h-Ki mice showed equivalent body weight, longevity, and reproductive patterns. Also, h-Ki mice showed normal hepatic function (reflected by serum ALT and AST activities) and liver lipid droplets (indicated by Oil Red O staining) (**Figure S4A-B**).

**Figure 2:**
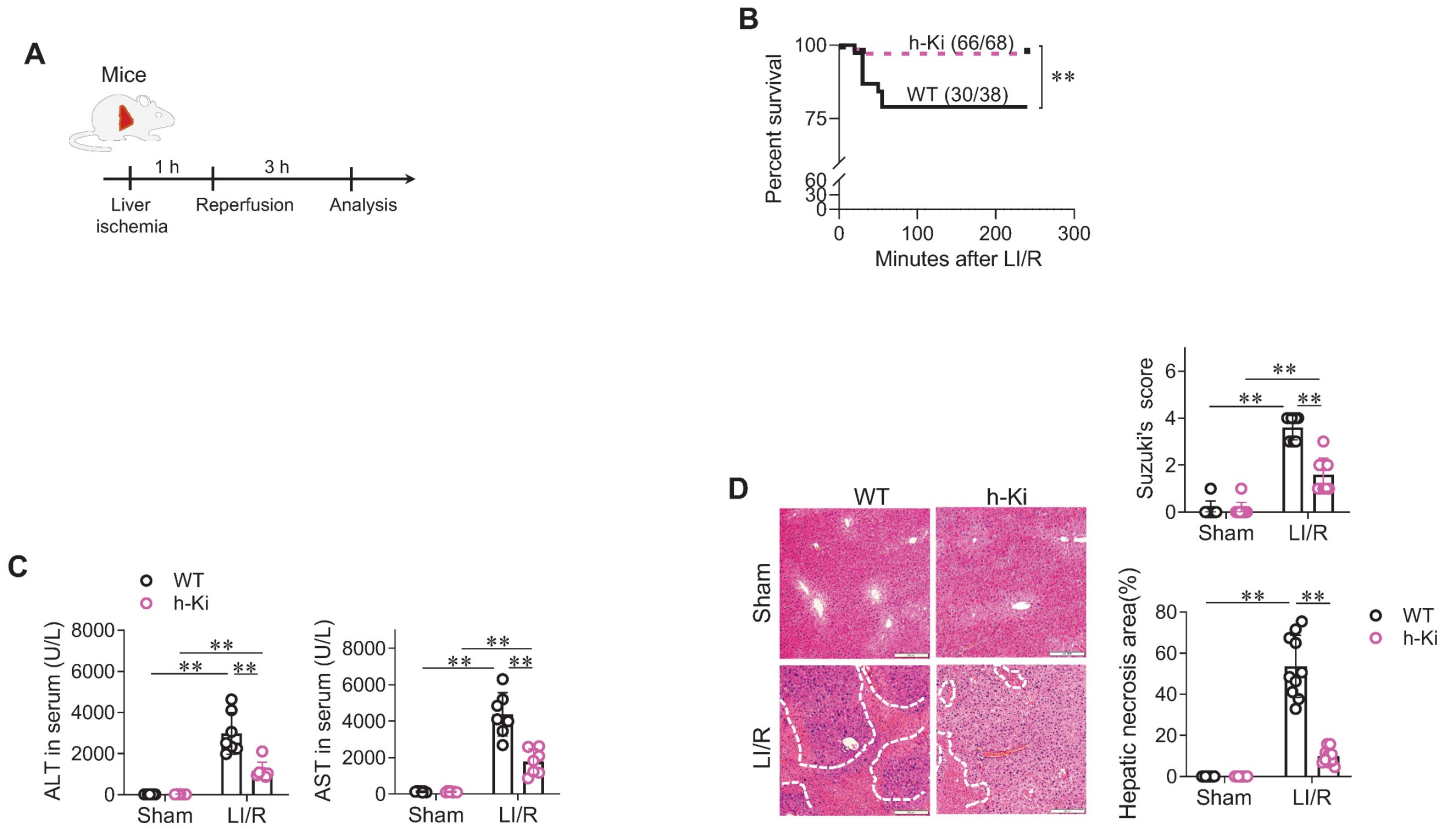
** H-Ki mice displayed attenuation of LI/R injury. A. Experimental protocol.** LI/R was induced in mice. **B. Mouse survival.** Mice mortality was recorded after LI/R. **P < 0.01 by log-rank test, n = 68 in h-Ki group and n = 38 in WT group. **C. Serum ALT and AST activities.** Serum was collected from mice after sham or LI/R for measuring ALT and AST activities. Data are mean ± SD, ** *P* < 0.01 by two-way ANOVA followed by Tukey's test. n = 7/group. **D. Histological examination.** After sham or LI/R, liver tissues were collected, paraffin-embedded sectioned, and HE stained. Suzuki's scoring was performed and hepatic necrosis areas were measured to indicate histological injury. Scale bar = 200 μm. Data are mean ± SD, ** *P* < 0.01 by two-way ANOVA followed by Tukey's test. n = 8 for sham-WT group and n = 10 for all the other groups.

To assess the functional significance of HSPA12A, LI/R injury was induced in WT and h-Ki mice (**Figure 2A**). After LI/R, h-Ki mice had higher survival rates than WT controls (**Figure 2B**). In addition, the LI/R-induced hepatic dysfunction, as indicated by increase of serum ALT and AST activities, was attenuated in h-Ki mice compared with WT controls (**Figure 2C**). In support of this, the LI/R-induced hepatic histological injury, as reflected by increase of Suzuki's scores and necrosis areas, were alleviated in h-Ki mice compared with WT controls (**Figure 2D**).

To assess whether HSPA12A is required for the protection against LI/R, HSPA12A knockout (Ko) mice were employed in the experiments. By contrast to h-Ki mice, Ko mice displayed exaggeration of the LI/R-triggered increases of serum ALT and AST activities compared to WT mice (**Figure S5A**). Moreover, Ko mice showed higher Suzuki's scores and larger necrosis areas then WT mice after LI/R (**Figure S5B**). In addition, a lower survival rate was found in Ko mice than WT mice following LI/R (**Figure S5C**).

### Hepatocyte HSPA12A suppresses macrophage chemotaxis to LI/R-exposed livers *in vivo* and to H/R-exposed hepatocytes *in vitro*

**Figure 3:**
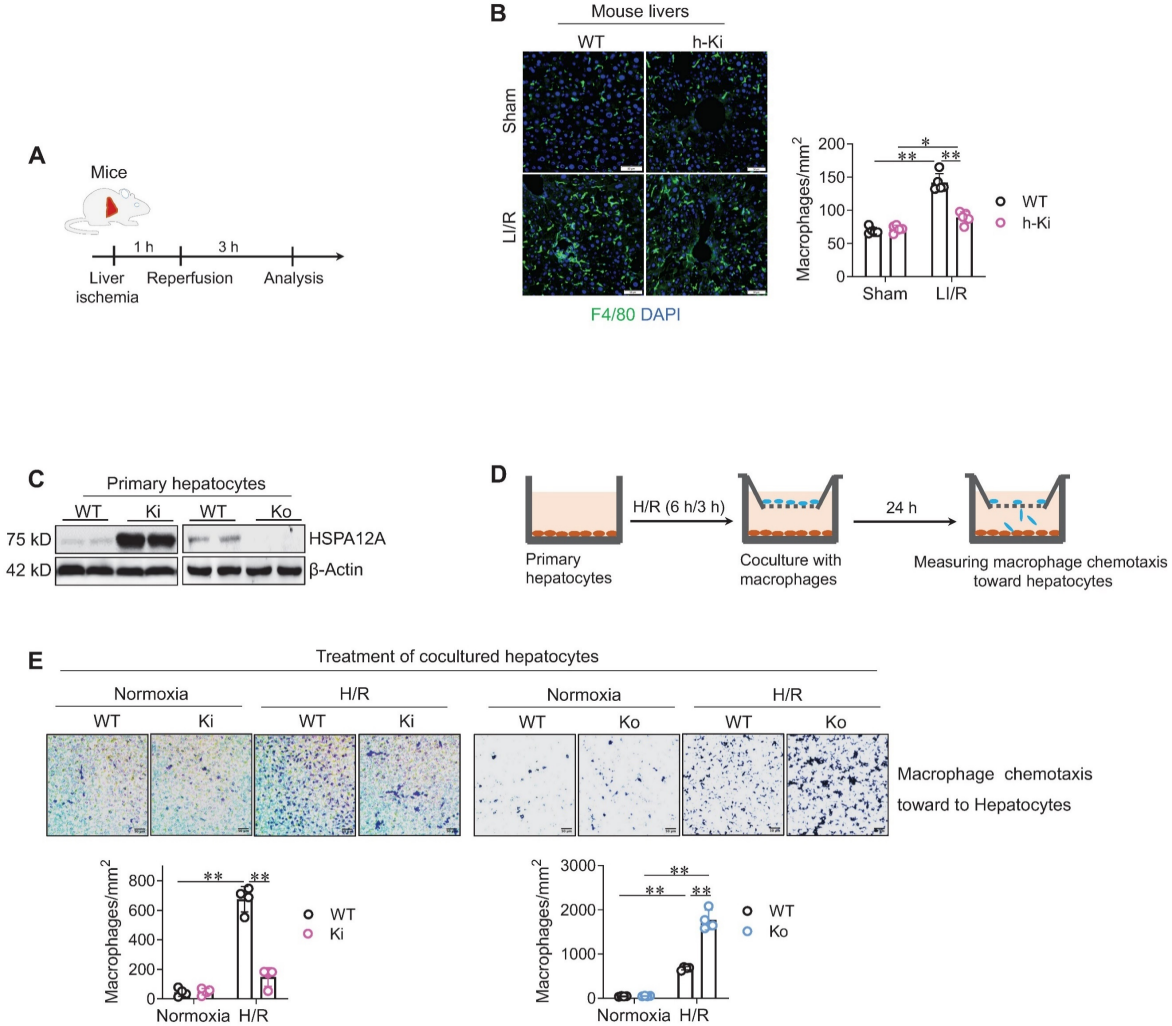
** Hepatocyte HSPA12A inhibited macrophage chemotaxis after LI/R. A-B. Mouse experiments.** LI/R was induced in mice (**A**). After LI/R, liver tissues were collected, cryosectioned, and immunestained for F4/80. DAPI was used to indicate nuclei (**B**). Scale bar = 50 μm. n = 5/group. **C-E. Cell experiments.** Primary hepatocytes with HSPA12A overexpression (Ki) or knockout (Ko) were isolated from livers of h-Ki or HSPA12A Ko mice. Hepatocytes from WT mice served as controls. HSPA12A expression was examined using immunoblotting (**C**). After exposed to H/R- or normoxia, hepatocytes were cocultured with primary macrophages in Transwell plates (**D**). After 24 h's coculture, macrophages chemotaxis toward hepatocytes was stained with crystal violet (**E**). Scale bar = 50 μm. n = 4/group. Data are mean ± SD, ** *P* < 0.01 and * *P* < 0.05 by two-way ANOVA followed by Tukey's test.

Hepatic macrophage chemotaxis and inflammatory activation are pivotal in the development of LI/R injury 4. We found that LI/R increased macrophage (F4/80^+^) recruitment in mouse livers; however, the LI/R-induced macrophage recruitment was lower in h-Ki mice than WT mice (**Figure 3A-B**), suggesting that hepatocyte HSPA12A inhibits macrophage chemotaxis to LI/R-exposed livers of mice.

To further verify this hypothesis,* in vitro* experiments were performed. To this aim, primary hepatocytes with HSPA12A overexpression (Ki) or knockout (Ko) were isolated from livers of h-Ki or HSPA12A-knockout mice, and primary hepatocytes from WT mice served as controls (**Figure 3C**). The hepatocytes were exposed to H/R for 6 h/3 h, followed by coculture with macrophages in Transwell plates under normal growth conditions (**Figure 3D**). After coculture for 24 h, more macrophages migrated toward H/R-hepatocytes than toward normoxia-hepatocytes (**Figure 3E**); however, compared with the migration toward H/R-WT hepatocytes, there was less macrophage migration toward H/R-Ki hepatocytes but more migration toward H/R-Ko hepatocytes (**Figure 3E**). Together, these findings indicate that overexpressing hepatocyte HSPA12A inhibits the chemotactic effects of H/R-hepatocytes on macrophage chemotaxis.

### Hepatocyte HSPA12A paracrinally inhibits macrophage inflammatory activation

**Figure 4:**
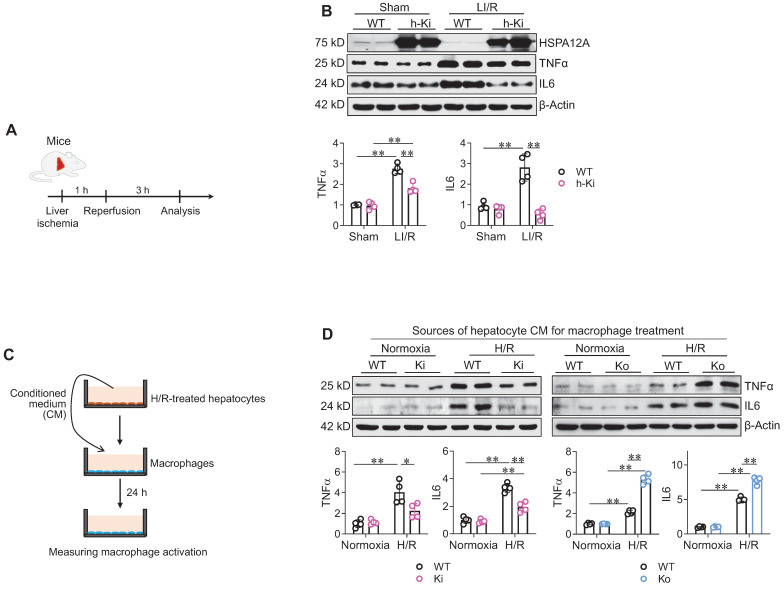
** Hepatocyte HSPA12A paracrinally inhibited macrophage inflammatory activation. A-B. Mouse experiments.** LI/R was induced in mice (**A**). Liver tissues were then collected to examine the indicated gene expression using immunoblotting (**B**). n = 4/group. **C-D. Cell experiments. (C)**. After exposed to H/R or normoxia, culture medium of primary hepatocytes was collected as hepatocyte conditioned medium (CM) and then added to macrophages (**D**). After treated with hepatocyte CM for 24 h, macrophages were collected to examine the indicated gene expression using immunoblotting. n = 4/group. Data are mean ± SD, ** *P* < 0.01 and * *P* < 0.05 by two-way ANOVA followed by Tukey's test.

Next, we examined the effect of hepatocyte HSPA12A on hepatic inflammatory responses after LI/R (**Figure 4A**). In mice, LI/R upregulated hepatic expression of inflammatory mediators (TNFα and IL6) compared to sham controls; however, the LI/R-induced upregulation of these mediators was attenuated in the livers of h-Ki mice compared to WT controls (**Figure 4B**).

We then investigated whether hepatocyte HSPA12A paracrinally modulates macrophage activation. To this aim, conditioned medium (CM) was collected from H/R-exposed hepatocyte cultures and then added to macrophage cultures for 24 h (**Figure 4C**). Compared with macrophages treated with CM from normoxia-WT hepatocytes, the macrophages treated with CM from H/R-WT hepatocytes had higher expression of TNFα and IL6 (**Figure 4D**). However, compared to macrophages treated with CM from H/R-WT hepatocytes, TNFα and IL6 expression was inhibited in macrophages treated with CM from H/R-Ki hepatocytes whereas promoted in macrophages treated with CM from H/R-Ko hepatocytes (**Figure 4D**). Moreover, compared with macrophages treated with CM from H/R-WT hepatocytes, the macrophages treated with CM from H/R-Ki hepatocytes were less spreading of morphology and those treated with CM from H/R-Ko hepatocytes showed higher expression of *Il6*, *Tnfα*, *Cd68*, and *Cd86* mRNA (**Figure S6A-C**). Together, these findings indicate that the H/R-exposed hepatocytes paracrinally activate an inflammatory phenotype in macrophages, while hepatocyte HSPA12A overexpression attenuates this response.

### Hepatocytes are injured directly by an H/R insult and are further injured by activated macrophages, and both injuries are alleviated by hepatocyte HSPA12A

Compared with normoxia, H/R exposure increased ALT and AST release from hepatocytes. However, this H/R-induced increase of ALT and AST release was attenuated in Ki hepatocytes but promoted in H/R-Ko hepatocytes when compare to WT cells (**Figure S7A**).

Next, we sought to determine whether activated macrophages can further damage H/R-exposed hepatocytes, and whether HSPA12A affects this process. To do this, we cocultured H/R-exposed hepatocytes with macrophages, as shown in **Figure S7B**. Notably, coculture with macrophages increased ALT and AST release from H/R-WT hepatocytes compared to those without macrophage coculture (**Figure S7B**). However, the macrophage coculture-induced increases of ALT and AST release was attenuated in H/R-Ki hepatocytes but promoted in H/R-Ko hepatocytes (**Figure S7B**).

Together, these data indicate that hepatocyte HSPA12A protects hepatocytes against H/R-induced injury as well as the further injury resulting from the activated macrophages.

### HSPA12A inhibits HMGB1 secretion from hepatocytes under LI/R-like conditions

**Figure 5:**
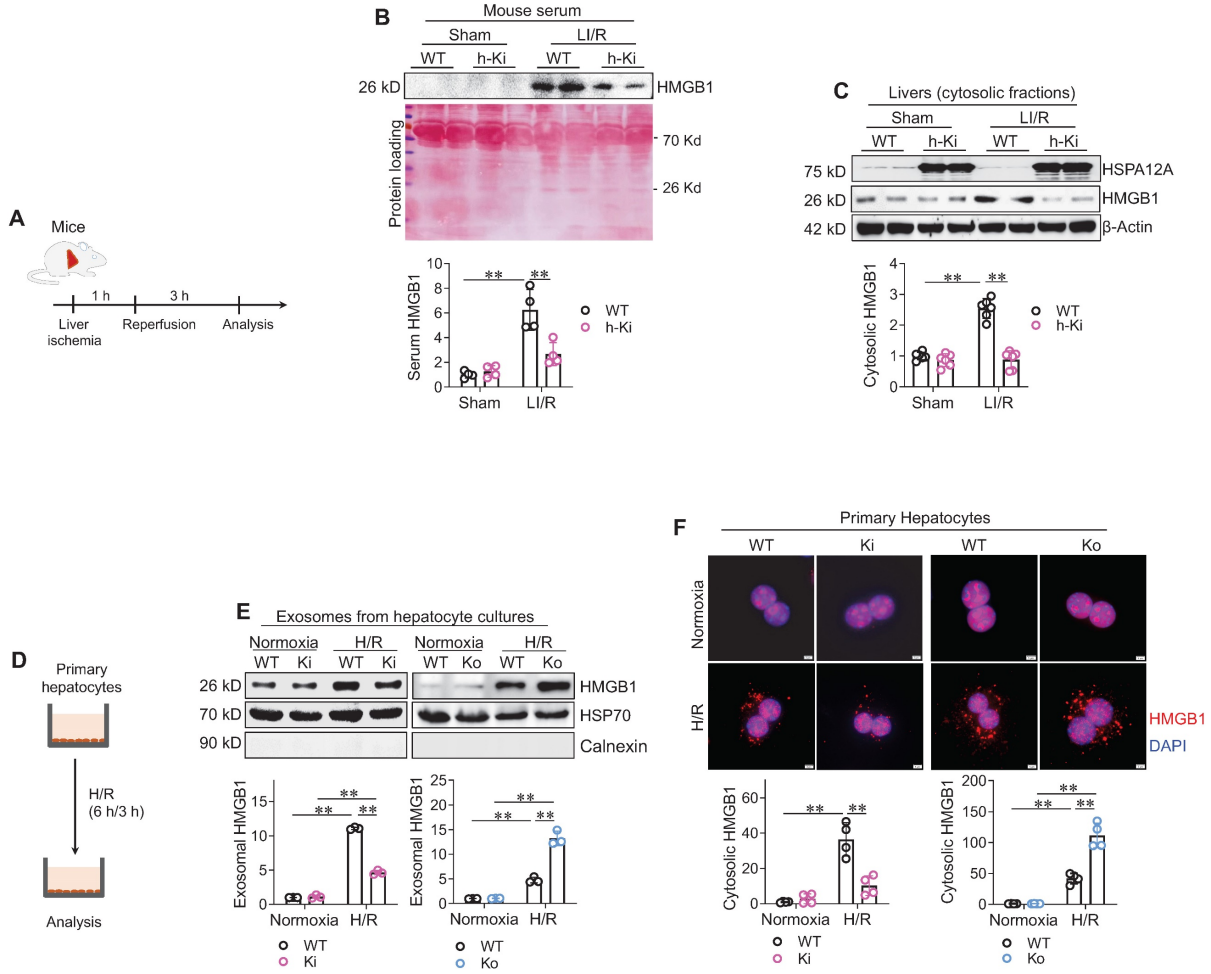
** Hepatocyte HSPA12A inhibited HMGB1 secretion from hepatocytes. A-C. Mouse experiments.** LI/R was induced in mice (**A**). Mouse serum was collected to examine HMGB1 protein levels using immunoblotting, and equal volume of serum separated on SDS-PAGE and stained with Ponceau S solution served as protein loading control (**B**). Also, liver tissues were collected, cytosolic fraction prepared, and immunoblotted for HMGB1 (**C**). n = 4/group (**B**) and n = 6/group (**C**). **D-F. Hepatocyte experiments.** H/R was induced in primary hepatocytes (**D**). After then, culture medium was collected, exosome isolated, and immunoblotted for HMGB1 (**E**). Blotting for HSP70 and Calnexin served as positive and negative markers, respectively (**E**). Also, primary hepatocytes were immunestained with HMGB1, and DAPI was used to stain nuclei (**F**). Scale bar = 5 μm. n = 3/group (**E**) and n = 4/group (**F**). Data are mean ± SD, ** *P* < 0.01 by two-way ANOVA followed by Tukey's test.

HMGB1 is a nuclear protein that triggers an inflammatory response when secreted into extracellular space, and the secreted HMGB1 is believed to mediate LI/R injury 8-10, 21. We then investigated whether HMGB1 release was involved in the hepatoprotection of HSPA12A (**Figure 5A**). Analyses of immunoblotting and enzyme-linked immunosorbent assay (ELISA) revealed that LI/R greatly increased HMGB1 levels in serum of WT mice; however, this LI/R-induced increase was alleviated in h-Ki mice (**Figure 5B, S8**). The cytosolic HMGB1 levels were next examined since HMGB1 translocates to the cytoplasm from the nucleus before secretion into the extracellular space 10. Consistent with the changes in serum HMGB1 levels, LI/R-induced increase of HMGB1 accumulation in cytosolic fraction was attenuated in livers of h-Ki mice compared to WT controls (**Figure 5C**).

To further explore the regulation of HSPA12A on HMGB1 secretion from hepatocytes, we measured HMGB1 levels in culture medium and cytoplasm of hepatocytes after H/R exposure (**Figure 5D**). HMGB1 was not detectable directly in culture medium (**Figure S9A-B**). Considering that HMGB1 is secreted into the extracellular space in exosomes 10, we isolated exosomes from culture medium. Exosomes from H/R-exposed hepatocyte medium had higher levels of HMGB1 than exosomes from normoxia-exposed hepatocyte medium (**Figure 5E**). However, this H/R-induced increase of exosomal HMGB1 was attenuated in Ki but promoted in Ko hepatocytes when compared to WT cells. Consistently, immunostaining showed that H/R exposure increased hepatocyte cytoplasmic HMGB1 accumulation, and this accumulation was attenuated in Ki hepatocytes but enhanced in Ko hepatocytes when compared to WT controls after H/R (**Figure 5F**). Thus, the data indicate that hepatocyte HSPA12A overexpression inhibits HMGB1 exosomal secretion from hepatocytes after exposure to LI/R-like conditions.

### HSPA12A supresses HMGB1 secretion from H/R-exposed hepatocytes to inhibit macrophage chemotaxis and activation

**Figure 6:**
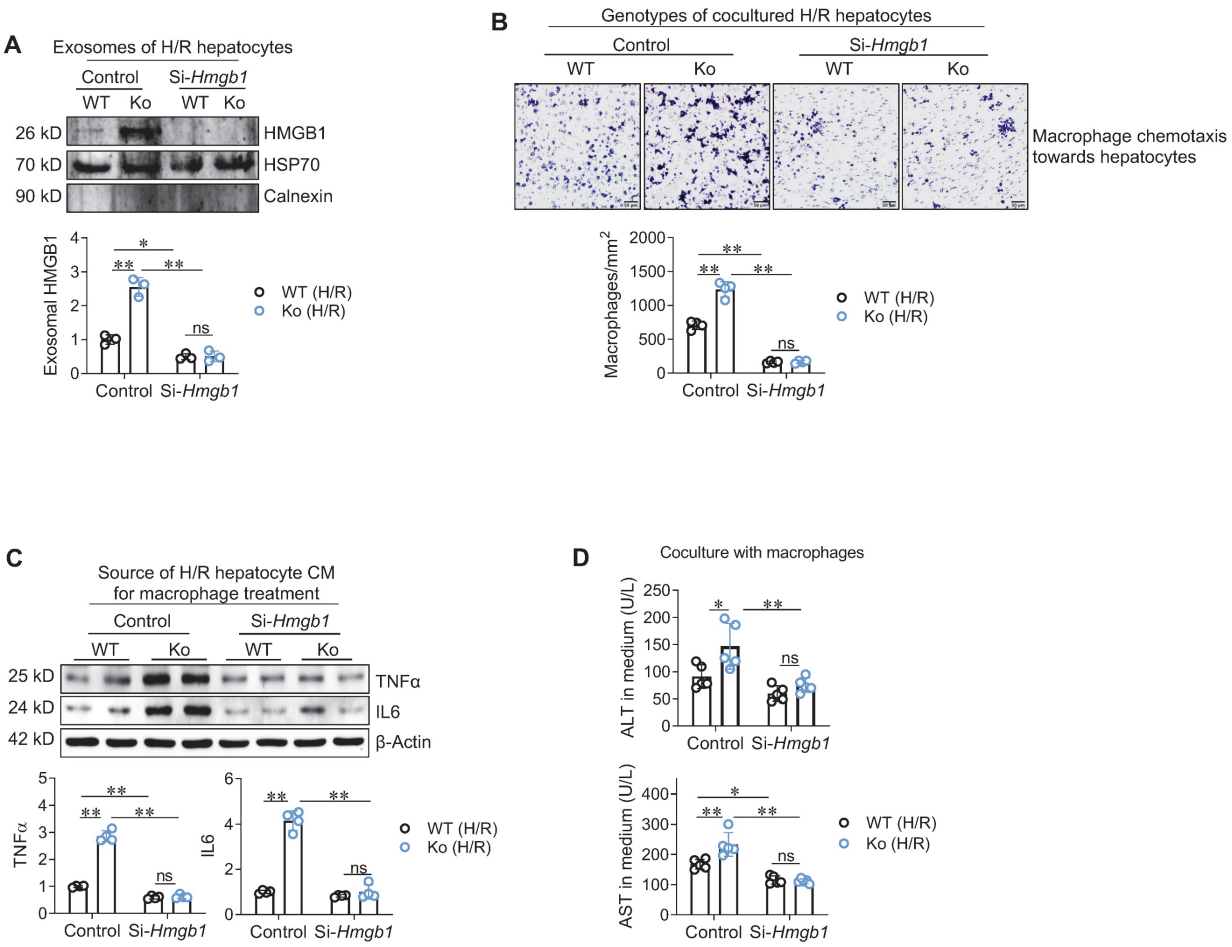
** Hepatocyte HSPA12A modulated HMGB1 secretion to paracrinally regulate macrophage chemotaxis and activation. A. HMGB1 knockdown decreased HMGB1 exosomal secretion of hepatocytes.** HMGB1 was knockdown in primary hepatocytes (Si-*Hmgb1*) and scramble RNA treatment served control. After exposed to H/R, culture medium was collected, exosome isolated, and immunoblotted for HMGB1. Blotting for HSP70 and Calnexin served as positive and negative markers, respectively. n = 3/group. **B. Hepatocyte HMGB1 knockdown inhibited macrophage chemotaxis.** After exposed to H/R, primary hepatocytes were cocultured with primary macrophages in Transwell plate. After coculture for 24 h, macrophage chemotaxis towards hepatocytes was stained with crystal violet. Scale bar = 50 μm. n = 4/group. **C. Hepatocyte HMGB1 knockdown inhibited macrophage activation.** After exposed to H/R, culture medium of primary hepatocytes was collected as hepatocyte conditioned medium (CM). The CM was then added to macrophages. After treated with hepatocyte CM for 24 h, macrophages were collected to examine the indicated gene expression using immunoblotting. n = 4/group. **D. Hepatocyte HMGB1 knockdown decreased ALT and AST leakage when coculture with macrophages.** Primary hepatocytes were exposed to H/R, followed by coculture with primary macrophages in Transwell plate. After coculture for 24 h, medium was collected for ALT and AST activity examination. n = 5/group. Data are mean ± SD, * *P* < 0.05 and ** *P* < 0.01 by two-way ANOVA followed by Tukey's test. ns, no significance.

Next, we determined whether hepatocyte-secreted HMGB1 mediates the effect of hepatocyte HSPA12A on macrophage chemotaxis and inflammatory activation. To this aim, HMGB1 was knocked-down in HSPA12A Ko hepatocytes by transfection with si-RNA targeting HMGB1 (Si-*Hmgb1*); hepatocytes transfected with scrambled si-RNA served as controls **(Figure S10A).** In normoxic condition, HMGB1 knockdown itself did not cause hepatocyte injury, as reflected by ALT and AST releases from hepatocytes **(Figure S10B)**. However, HMGB1 knockdown reversed the promotion of exosomal HMGB1 secretion from H/R-Ko hepatocytes (**Figure 6A**). Moreover, hepatocyte HMGB1 knockdown abolished the promoted effects of H/R-Ko hepatocytes on macrophage chemotaxis and macrophage expressing TNFα and IL6, respectively, compared with the scramble H/R-Ko hepatocytes (**Figure 6B-C**). In addition, HMGB1 knockdown in hepatocytes abolished the elevations in ALT and AST release from H/R-Ko hepatocytes either with or without macrophage coculture (**Figure 6D, S11A-B**). Together, the data suggest that HSPA12A suppresses hepatocyte HMGB1 secretion to inhibit the effects of H/R-hepatocytes on macrophage chemotaxis and activation.

### HSPA12A suppresses HMGB1 lactylation to inhibit its secretion from H/R-exposed hepatocytes

**Figure 7:**
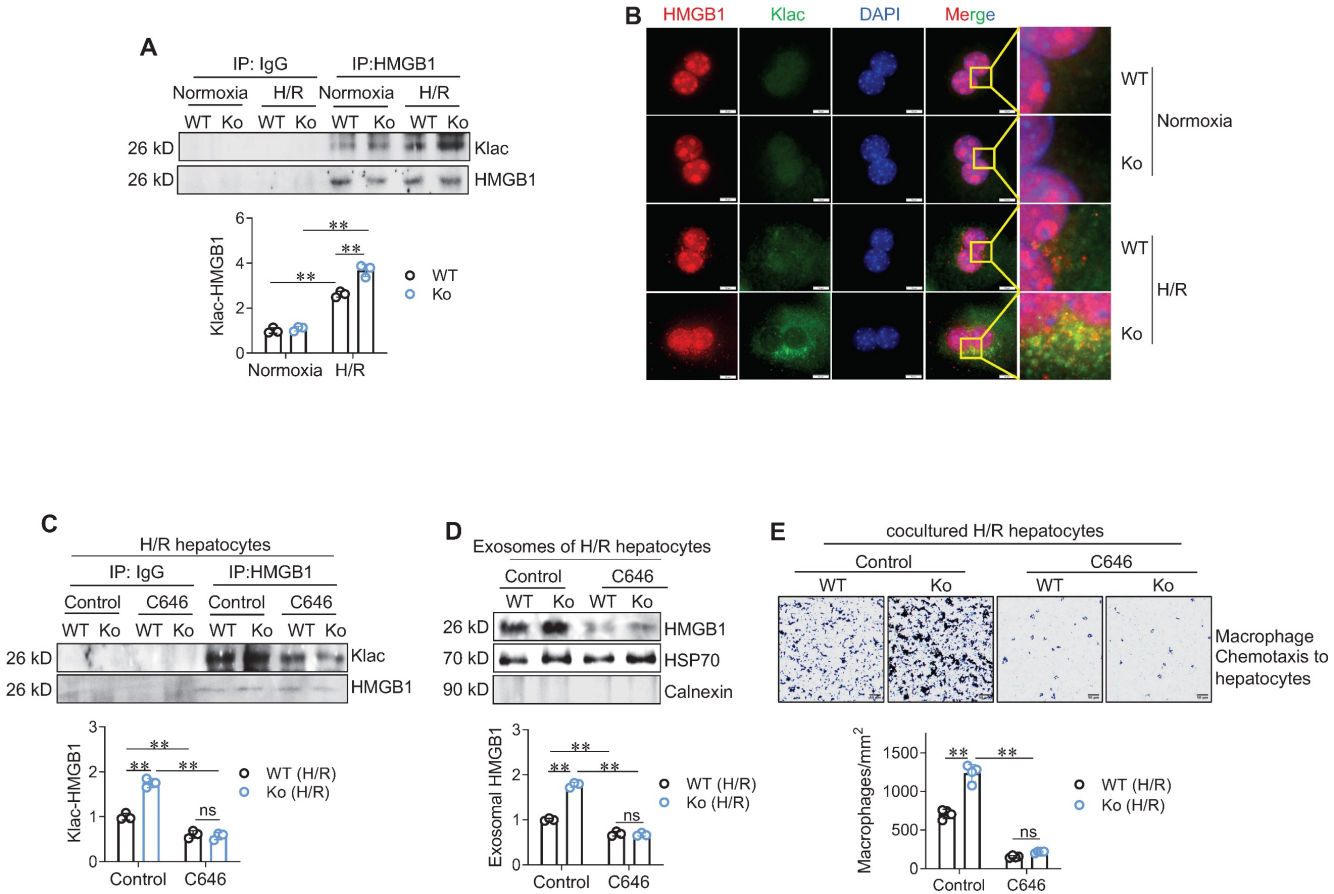
** HSPA12A modulated hepatocyte HMGB1 lactylation and exosomal secretion to paracrinally regulate macrophage chemotaxis. A. Klac-HMGB1 levels in hepatocytes.** After exposed to H/R or normoxia, primary hepatocytes were immunoprecipitated with anti-HMGB1 antibody followed by immunoblotting for Klac and HMGB1. n = 3/group. **B. Colocalization of Klac and HMGB1 in cytoplasm of hepatocytes.** After exposed to H/R or normoxia, primary hepatocytes were immunestained with Klac and HMGB1. DAPI was used to counter stain nuclei. Note that the H/R-induced Klac-HMGB1 colocalization was promoted in cytoplasm of HSPA12A Ko hepatocytes. Scale bar = 10 μm. n = 3/group. **C. C646 decreased HMGB1 lactylation levels.** Primary hepatocytes were exposed to H/R in the presence or absence of C646. After then, hepatocytes were immunoprecipitated with anti-HMGB1 antibody followed by immunoblotting for Klac and HMGB1. n = 3/group. **D. C646 decreased exosomal HMGB1 secretion**. Primary hepatocytes were exposed to H/R in the presence or absence of C646. After then, culture medium was collected, exosome isolated, and immunoblotted for HMGB1. Blotting for HSP70 and Calnexin served as positive and negative markers, respectively. n = 3/group. **E. C646 abolished the enhanced chemotactic effects of Ko hepatocytes on macrophage chemotaxis.** Primary hepatocytes were exposed to H/R in the presence or absence of C646, followed by coculture with primary macrophages in Transwell plate. After coculture for 24 h, macrophage chemotaxis was crystal violet stained and quantified. Scale bar = 50 μm. n = 4/group. Data are mean ± SD, ** *P* < 0.01 by two-way ANOVA followed by Tukey's test. ns, no significance.

Next, we asked how HSPA12A modulates HMGB1 secretion from hepatocytes. In macrophages during sepsis, lactylation mediates HMGB1 cytosolic accumulation for the subsequent exosomal release 10. We then examined HMGB1 lactylation levels in hepatocytes according to previous methods 10. Intriguingly, H/R-exposed hepatocytes displayed increased lactylation (Klac) levels in HMGB1 immunoprecipitates compared to normoxia-exposed cells; however, this H/R-increased Klac-HMGB1 levels were promoted in Ko hepatocytes (**Figure 7A**) but attenuated Ki hepatocytes (**Figure S12A**) when compared to their WT controls. Consistently, the H/R-induced increase of Klac-HMGB1 colocalization in the cytoplasm of hepatocytes was enhanced in Ko hepatocytes (**Figure 7B**) but decreased in Ki hepatocytes (**Figure S12B**) than their WT controls.

To determine the role of HMGB1 lactylation in HSPA12A-modulated HMGB1 secretion from hepatocytes, we treated H/R-Ko hepatocytes with the p300 inhibitor C646 to suppress HMGB1 lactylation 10. C646 treatment reversed the increases of Klac-HMGB1 levels (**Figure 7C**) and exosomal HMGB1 secretion (**Figure 7D**) of H/R-Ko hepatocytes. Also, the H/R-Ko hepatocytes-induced increase of macrophage chemotaxis was abolished by C646 treatment (**Figure 7E**). Together, these findings suggest that HSPA12A inhibits HMGB1 exosomal secretion by inhibiting HMGB1 lactylation in H/R-exposed hepatocytes.

### HSPA12A decreases glycolysis-derived lactate to inhibit HMGB1 lactylation in H/R-exposed hepatocytes

**Figure 8:**
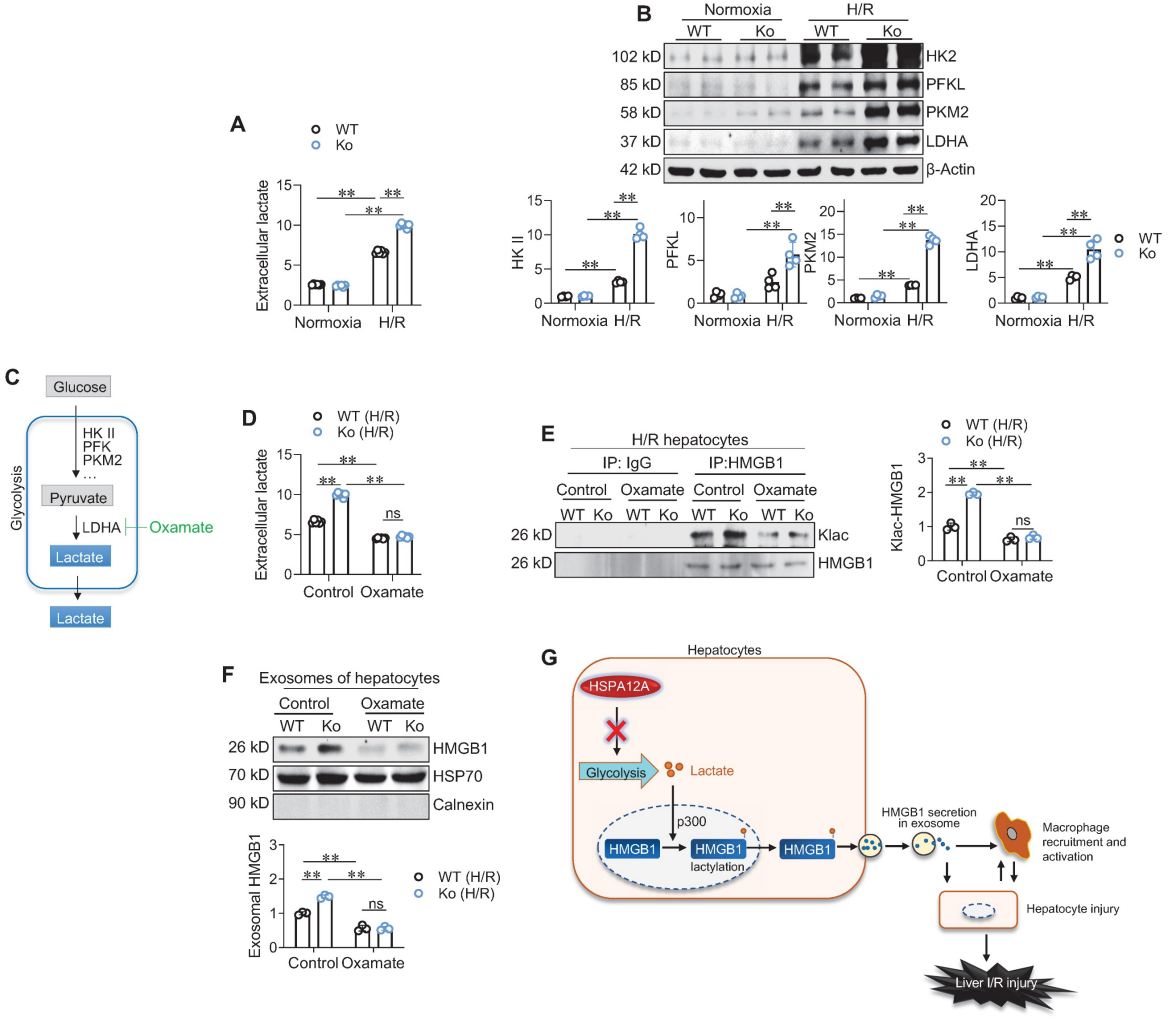
** HSPA12A inhibited glycolysis-derived lactate to suppress HMGB1 lactylation and exosomal secretion of H/R-exposed hepatocytes. A. Extracellular lactate.** After exposed to normoxia or H/R, culture medium of primary hepatocytes was collected for measuring lactate levels. n = 6/group. **B. Expression of glycolysis-related genes.** After exposed to normoxia or H/R, primary hepatocytes was collected for measuring the indicated gene expression using immunoblotting. n = 4/group. **C. Brief scheme of glycolytic process and inhibition.** Glucose is uptaken into cells, converted to pyruvate by enzymes (HK II, PFK and PKM2) and followed by converting to lactate by LDHA, and finally lactate is exported to extracellular space. For inhibiting lactate production, Oxamate was used to inhibit LDHA activity. **D. Oxamate inhibited lactate production.** Primary hepatocytes were exposed to H/R in the presence or absence of Oxamate. After then, culture medium was collected for measuring lactate levels. n = 6/group. **E. Oxamate inhibited HMGB1 lactylation.** Primary hepatocytes were exposed to H/R in the presence or absence of Oxamate. After then, hepatocytes were immunoprecipitated with anti-HMGB1 antibody followed by immunoblotting for Klac and HMGB1. n = 3/group. **F. Oxamate inhibited exosomal HMGB1 secretion.** Primary hepatocytes were exposed to H/R in the presence or absence of Oxamate. After then, exosomes were isolated from culture medium and immunoblotted for HMGB1. Blotting for HSP70 and Calnexin served as positive and negative markers, respectively. n = 3/group. **G. Mechanistic scheme.** By suppressing glycolysis-derived lactate production in hepatocytes, HSPA12A inhibits HMGB1 lactylation and exosomal secretion of hepatocytes, thereby disrupts the “hepatocyte damage - macrophage chemotaxis/activation - hepatocyte damage” inflammatory toxic cycle, and ultimately leads to protection against LI/R injury. Data are mean ± SD, ** *P* < 0.01 by two-way ANOVA followed by Tukey's test. ns, no significance.

Finally, we sought to identify how HSPA12A inhibits HMGB1 lactylation in H/R-exposed hepatocytes. Lactylation is the process of adding lactyl groups to proteins using glycolysis-derived lactate 16. Glycolysis is the metabolic process of converting glucose into pyruvate, which can subsequently be converted to lactate, and thus lactate exportation into the extracellular space is considered a readout of glycolytic flux 19, 22. We found that H/R exposure increased extracellular lactate levels compared to normoxia controls; however, this increase was further enhanced in Ko hepatocytes compared with WT controls (**Figure 8A**). In supporting this, the H/R-upregulated expression of glycolysis-genes (HK2, PKM2, and LDHA) was enhanced in Ko hepatocytes compared with WT controls (**Figure 8B**). By contrast, Ki hepatocytes displayed prevention of the H/R-induced increase of extracellular lactate levels and upregulation of glycolysis-related gene expression when compared WT cells (**Figure S13A-C**). Therefore, HSPA12A inhibit the H/R-induced increase of glycolysis-derived lactate in hepatocytes.

To determine the role of glycolysis-derived lactate in HSPA12A-regulated HMGB1 lactylation, H/R-exposed hepatocytes were treated with the glycolysis inhibitor Oxamate to block lactate generation (**Figure 8C**). As expected, Oxamate reversed the increase of extracellular lactate levels of H/R-Ko hepatocytes (**Figure 8D**). Remarkably, Oxamate reversed the increase of Klac-HMGB1 levels in H/R-Ko hepatocytes (**Figure 8E**). Importantly, the increased exosomal HMGB1 secretion levels of H/R-Ko hepatocytes were also reversed following Oxamate treatment (**Figure 8F**). Together, these findings suggest that HSPA12A negatively regulates HMGB1 lactylation and exosomal secretion through modulating glycolysis-generated lactate in H/R-exposed hepatocytes.

## Discussion

In this study, we found that the increased macrophage chemotaxis in liver was accompanied by decreased HSPA12A expression in hepatocytes following LI/R, whereas overexpression of hepatocyte HSPA12A attenuated LI/R-induced macrophage chemotaxis and liver injury in mice. This protective effect of hepatocyte HSPA12A was mediated by decreasing glycolysis-generated lactate, which inhibited the subsequent cascade of hepatocyte HMGB1 lactylation and secretion, macrophage chemotaxis and activation, and inflammation-related hepatocyte damage (**Figure 8G**). These findings suggest that hepatocyte HSPA12A is a potential therapeutic target for clinical management of LI/R injury.

LI/R injury is a serious clinical complication triggered by liver resection and transplantation, sepsis/septic shock, trauma, hemorrhagic shock, and cardiac ischemia 1. Since it can cause acute and chronic hepatic dysfunction, liver graft rejection, and hepatic cirrhosis, LI/R injury is a critical prognostic determinant in patients 2-4. However, interventions to mitigate LI/R injury are still lack. Mounting studies demonstrate that LI/R injury is characterized by a local, sterile inflammatory response driven by innate immunity and hepatocyte damage 4, 6, 23. This sterile inflammatory response involves multiple cells. First, ischemic insult damages hepatic cells; these then secrete DAMPs, especially HMGB1, to chemoattract macrophages and other immune cells from the circulation after reperfusion. Following activation by DAMPs, the chemoattracted macrophages release inflammatory cytokines causing hepatocyte death and also releasing chemotactic cytokines recruiting more circulating immune cells to LI/R-exposed livers. Thus, the interaction between LI/R-injured hepatocytes and macrophage chemotaxis/activation forms a toxic inflammatory cycle, and disrupting this cycle by inhibiting the effect of LI/R-affected hepatocytes on macrophage chemotaxis could therefore limit LI/R injury.

Indeed, this hypothesis is supported by the present findings. First, we observed that macrophages were chemoattracted to LI/R livers of mice *in vivo* as well as to H/R hepatocytes *in vitro*. Second, h-Ki mice displayed attenuation of the LI/R-induced macrophage chemotaxis, hepatic dysfunction, and animal mortality. Finally, coculture with macrophages aggravated the H/R-induced hepatocyte injury, and this aggravation was attenuated by hepatocyte HSPA12A overexpression. Collectively, our findings indicate that hepatocyte HSPA12A mitigated LI/R injury through disrupting the inflammatory cycle between LI/R-affected hepatocyte and macrophage chemotaxis/inflammatory activation.

HSPA12A is a heat shock protein that was identified in 2003 and classified as a distant and atypical member of the HSP70 family 17. Heat shock proteins are an evolutionarily conserved superfamily comprising a group of structurally unrelated subfamilies, including HSPA/HSP70, HSPB/HSP27, HSPC/HSP90, HSPH/HSP110, and NDAJ/HSP40 24, 25. Of these, HSP27, HSP70, and HSP90 are involved in LI/R injury 26, 27. However, the unsuccessful translation of these HSPs to clinical treatment of LI/R injury indicates that more HSPs may be involved. In this study, we found that HSPA12A was downregulated in hepatocytes when macrophages were chemoattracted to livers following LI/R, suggesting a possible involvement of hepatocyte HSPA12A in LI/R-induced macrophage chemotaxis and hepatic injury. Indeed, hepatocyte overexpression attenuated the LI/R-induced macrophage recruitment and hepatic injury in mice *in vivo* as well as the H/R hepatocyte-induced macrophage chemotaxis and activation *in vitro*. Moreover, the further damage of H/R hepatocytes caused by activated macrophages was also abolished by hepatocyte HSPA12A overexpression. Taken together, these results provide strong evidence that HSPA12A is a novel hepatoprotective HSP protein against LI/R injury.

Nuclear HMGB1 is a DNA-binding protein that regulates gene expression under normal conditions; however, extracellular HMGB1 is a DAMP molecule that acts as both an inflammatory cytokine and chemotactic mediator. Indeed, inhibiting HMGB1 release from macrophages or suppressing HMGB1 activity with neutralizing antibodies have protective roles against LI/R injury 14, 28. In this study, we found that circulating HMGB1 level was increased in mice after LI/R, and hepatocytes secreted more HMGB1 after H/R. In addition, knockdown of HMGB1 in hepatocytes reversed the enhanced chemotaxis and activation of macrophages induced by H/R-HSPA12A Ko hepatocytes. Together, our findings suggest that hepatocyte HSPA12A inhibits macrophage chemotaxis and inflammatory activation by suppressing hepatocyte HMGB1 secretion after LI/R.

The secretion of HMGB1 involves two steps: shuttling from the nucleus to the cytoplasm and followed by exosomal secretion into the extracellular space 7, 10. The shuttling of HMGB1 from the nucleus to the cytoplasm is mediated by several types of post-translational modification, such as acetylation and deacetylation 7. Intriguingly, we found that in macrophages during sepsis, lactylation of HMGB1 mediates its exosomal secretion 10. However, it is unknown whether lactylation mediates HMGB1 secretion from hepatocytes, and how HMGB1 lactylation is regulated after LI/R. Lactylation is a recently identified post-translational modification by p300 using glycolysis-derived lactate as a substrate. In this study, we found the following results. (1) HSPA12A inhibited the glycolysis-derived lactate generation and HMGB1 lactylation in hepatocytes following exposure to H/R; (2) Inhibiting HMGB1 lactylation in H/R-exposed hepatocytes, either by blocking lactate generation or by using a p300 inhibitor, suppressed HMGB1 cytosolic accumulation and the subsequent exosomal secretion; (3) Remarkably, inhibition of HMGB1 lactylation ablated the promoted effects of H/R-Ko hepatocytes on macrophage chemotaxis induced by. Together, our findings indicate that hepatocyte HSPA12A inhibits macrophage chemotaxis by suppressing glycolysis-mediated HMGB1 lactylation and exosomal secretion of H/R-exposed hepatocytes.

## Conclusion

This study demonstrates that hepatocyte HSPA12A protected against LI/R injury in mice. This protective effect of hepatocyte HSPA12A was mediated by inhibiting glycolysis-mediated HMGB1 lactylation and exosomal secretion from hepatocytes, by which suppressed macrophage chemotaxis and inflammatory activation. Our data suggest that targeting hepatocyte HSPA12A may be useful for the management of LI/R injury in patients.

## Materials and Methods

### Reagents

Collagenase Type IV was from Worthington biochemical Corporation (Lakewood, NJ). Percoll density gradient media was from GE Healthcare (Uppsala, Sweden). Protein A-Agarose was from Santa Cruz Biotechnology (Dallas, TX). Peroxidase-, Cy3-, and 488-conjugated secondary antibody were from Jackson lmmunoResearch (West Grove, PA). Trizol reagent was from Life Technology (Carlsbad, CA). SYBR Green Master and bovine serum albumin (BSA) was from Roche (Basel, Switzerland). High-sig ECL western blotting substrate was from Tanon (Shanghai, China). Oil Red O (O0625; Sigma-Aldrich, Saint Louis, MO). Lactate assay kit was from Jiancheng Biotech (Nanjing, China). ELISA kit for HMGB1 measurement was from Cloud-Clone Corp. (Wuhan, China). DMEM medium and fetal bovine serum (FBS) was from Gibco (Shelton, CT). RPMI 1640 medium were from Biological Industries (Kibbutz Beit Haemek, ISRAEL). C646 and Oxamate (Oxa) were obtained from MedChemExpress (Monmouth Junction, NJ) and Selleck Chemicals (Boston and Houston) respectively. The antibodies were listed in **Supplementary Table S1.**

### Animals

#### Creation of knockin mice with hepatocyte-specific overexpression of HSPA12A (h-Ki)

H-Ki mice was created by two steps: conditional HSPA12A knockin mice were created and crossed with Alb-Cre transgenic mice. The conditional HSPA12A knockin mice were created using CRISPR/Cas9 system. Briefly, one sgRNA-targeting the near sequence of inserted site constructed and transcribed *in vitro*, and the donor vector with the inserted fragment was designed and constructed *in vitro*. Then, Cas9 mRNA, sgRNA, and the donor were co-injected into zygotes followed by transplanting these zygotes into the oviduct of pseudo-pregnant ICR females at 0.5 dpc. After 19~21 days of transplantation, F0 mice were born, identified by PCR and tail DNA sequencing, and crossed with wild type mice to build up heterozygous conditional *Hspa12a* knockin mice. To obtain knockin mice with hepatocyte-specific overexpression of HSPA12A, conditional HSPA12A knockin mice were crossed with Alb-Cre transgenic mice. The construction and breeding strategies were shown in **Figure S2A-B**, and genotyping was shown in **Figure S2C**.

#### HSPA12A knockout (Ko) mice

HSPA12A Ko mice were obtained by crossing conditional HSPA12A knockout mice with EIIa-Cre transgenic mice as described in our previous studies 18, 25, 29.

All mice were with C57B/L6 genetic background. Mice were randomly assigned to all analyses. Investigators were blinded to the histological analysis. Investigators involved in animal handling, sampling, and raw data collection were not blinded. The mice were bred at the Model Animal Research Center of Nanjing University and were maintained in the Animal Laboratory Resource Facility of the same institution. All experiments conformed to the Guide for the Care and Use of Laboratory Animals published by the US National Institutes of Health (NIH Publication, 8th Edition, 2011). The animal care and experimental protocols were approved by Nanjing University's Committee on Animal Care. All experiments conformed to international guidelines on the ethical use of animals.

### LI/R in mice

LI/R was induced in male mice (8-10-week-old) by an established model of segmental (70%) hepatic ischemia/reperfusion 30, 31. Briefly, mice were anaesthetized with inhalation of 1.5-2% isoflurane, the arterial and portal venous blood supply to the cephalad lobes was occluded by an atraumatic clip for 60 min, and then the clip was removed for blood reperfusion. Sham-operated mice underwent the same procedure, but without vascular occlusion. For analgesia, buprenorphine (0.05 mg/kg) was administrated subcutaneously, and for blood sampling and tissue collection, mice were sacrificed by overdose anaesthesia (pentobarbital sodium 150 mg/kg intraperitoneal injection) and cervical dislocation according to our previous studies 32. For mortality recording, mice were checked every 20 min after LI/R.

### Biochemical analysis

Activities of AST and ALT in mouse serum and cell culture medium were measured using a Beckman Coulter AU5800 Chemistry System analyzer (Brea, CA). Levels of lactate in mouse serum were measured by a blood gas analyzer (iSTAT analyzer MN:300, Abbott Park, IL), and lactate levels in cell culture medium were examined using the assay kit. The levels of HMGB1 in the serum of mice were examined using an ELISA kit according to the manuals from the manufacturer.

### Cell Culture, H/R, and other treatment

#### Isolation and culture

Primary hepatocytes and primary liver macrophages were isolated from 8-10-week-old mice using our previous methods 33. Briefly, livers were perfused with Hank's buffered salt solution followed by digested with 0.06% collagenase type IV. After centrifugation of the digestions at 50 × g for 3 min, the pellets containing hepatocytes were plated on a collagen-coated plates, and the supernatants were further subjected to 25%-50% Percoll gradient centrifugation for macrophage collection. Primary hepatocytes were grown in DMEM containing 10% FBS and 0.01 mM dexamethasone, and primary macrophages were cultured in RPMI 1640 medium with 10% FBS. Raw264.7 macrophages were grown in the same condition with primary macrophages.

#### HSPA12A overexpression (Ki) or knockout (Ko)

Ki and Ko hepatocytes were isolated from livers of h-Ki mice and HSPA12A Ko mice, respectively. Primary hepatocytes from WT mice served as controls.

#### HMGB1 knockdown (Si-*Hmgb1*)

To knockdown HMGB1 expression, primary hepatocytes were transfected with siRNA that targeting mouse *Hmgb1* mRNA for 24 h. The primary hepatocytes transfected with scrambled RNA served as controls. The siRNA sequences were shown in **Supplementary Table S2.**

#### Hypoxia/reoxygenation (H/R)

To induce hypoxia, primary hepatocytes were changed to serum- and glucose-free DMEM medium and incubated in 94% N_2_, 5% CO_2_, and 1% O_2_ in a humidified incubator at 37°C. After 6 h of hypoxia, hepatocytes were changed back to normal growth medium and normal incubation condition for reoxygenation.

#### Effects of hepatocyte HSPA12A on macrophage chemotaxis

A Transwell system (Corning Inc, New York, NY) was used in this analysis 34. Briefly, primary hepatocytes that growing on lower chamber were exposed to H/R for 6 h/3 h, followed by coculture with primary macrophages that growing on insert membrane with 8 μm pore size. The coculture was conducted in normal culture medium and normal incubation conditions. After coculturing for 24 h, the migrated macrophages on the backside of inserts were fixed with 4% paraformaldehyde, stained with crystal violet (0.5%), and quantified in four randomly selected areas at a magnification of 100 × of each sample using Cellsens Dimention 1.15 software (Olympus, Tokyo, Japan).

#### Paracrine effects of hepatocyte HSPA12A on macrophage activation

Primary hepatocytes were exposed to H/R for 6 h/3 h, and the culture medium was collected as conditioned medium (CM). The hepatocyte CM was then added to Raw264.7 macrophages. After incubation with CM for 24 h, Raw264.7 macrophages were collected for analyzing expression of the indicated genes using immunoblotting and real-time PCR.

#### Oxamate or C646 treatment

For inhibiting lactate generation form glycolysis, hepatocytes were treated with Oxamate (10 mM). For inhibiting lactylation, hepatocytes were treated with a p300 inhibitor C646 (5 μM).

### Exosome preparation and characterization

Exosomes are prepared from cell culture medium using ultracentrifugation methods according to previous studies 35, 36. Briefly, culture medium was centrifuged at 300 × g for 10 min to eliminate dead cells and at 2,000 × g for 10 min and followed by 10,000 × g for 30 min to eliminate cell debris. The supernatants were then ultracentrifuged at 100,000 × g for 70 min to pellet the small vesicles that correspond to exosomes. After washing with phosphate-buffered saline and ultracentrifugation again at the same speed, pellets were resuspended in radio immunoprecipitation assay buffer (Beyotime Biotech, China) for 3 min on ice to prepare exosomal lysates. All centrifugations were conducted at 4 ºC. The presence of exosome marker HSP70, and the absence calnexin was confirmed by western blot according to previous methods 10.

### Histological analysis and immunofluorescence staining

Histology of mouse livers was examined using HE staining as our previous methods 33. After LI/R for 1 h/3 h, hepatic tissues were collected, prepared for paraffin-embedded sectioning, and stained with HE solution. The severity of L/IR injury was assessed blindly and graded according to Suzuki's criteria on a scale from 0 to 4. No necrosis, congestion, or centrolobular ballooning is given a score of 0, while severe congestion and greater than 60% lobular necrosis is given a value of 4 37, 38. The scoring was performed on four randomly selected areas of each sample using Cellsens Dimention 1.15 software (Olympus, Tokyo, Japan). The hepatic necrosis areas were measured on HE stained, paraffin-embedded liver sections using Image J software (National Institutes of Health, Bethesda, MD, USA).

Immunofluorescence staining of frozen liver sections or primary hepatocytes was performed as our previous methods 25, 33. Briefly, after incubation with the indicated primary antibodies overnight at 4 ºC, Cy3- or FITC-conjugated appropriate secondary antibodies were added to the samples to visualize the staining. DAPI reagent was used to counterstain the nuclei. The staining was observed and quantified in eight to ten randomly selected areas of each sample using a fluorescence microscope with Cellsens Dimention 1.15 software (Olympus, Tokyo, Japan).

### Oil Red O Staining of Liver

Oil Red O was used to stain lipid droplets according to our previous method 33. Briefly, frozen liver sections were fixed with 4% paraformaldehyde for 30 min, then incubated in 60% isopropanol for 2 min at room temperature, followed by Oil Red O staining buffer for 15 min. The staining was observed using a microscope (Olympus, Tokyo, Japan).

### Immunoblotting and immunoprecipitation-immunoblotting

Proteins were prepared from livers or cells for immunoblotting according to our previous methods 18, 33. To control for lane loading, the membranes were probed with anti-β-Actin or GAPDH antibody.

HMGB1 lactylation was examined by immunoprecipitation-immunoblotting according to previous methods 10. Briefly, hepatocytes were collected for protein extraction. Aliquots of equal volume and protein content were precipitated with anti-HMGB1 antibody, and the immunoprecipitates were immunoblotted for HMGB1 and L-Lactyl Lysine.

### Quantitative real-time PCR

Quantitative real-time PCR was performed as described previously 18. Briefly, Total RNA was isolated for cDNA synthesis. The expressions of indicated genes were estimated by real-time PCR using the SYBR Green Master. The PCR results of *Actin* served as internal controls. The primers used for PCR are listed in **Supplementary Table S3**.

### Microarray analysis

Microarray based transcription profiles were analyzed in liver tissues after LI/R (Shanghai Applied Protein Technology, China). Data were quantile normalized, summarized using robust multichip analysis and adjusted for probe sequence. A log2 conversion and a baseline transformation to the median of all samples were carried out. Expression of *Hsp* mRNA levels was visualized using heatmaps and histogram performed using R software version 4.0.5 and GraphPad Prism 7 (GraphPad Software, CA).

### Statistical analysis

Data are expressed as the mean ± standard deviation. Groups were compared using Student's two-tailed unpaired t test, log-rank test, or using one-way and two-way ANOVA followed by Tukey's post-hoc test. A *P* value of *<* 0.05 was considered significant.

## Supplementary Material

Supplementary figures.
