# Supplementary material for: Hepatocyte HSPA12A inhibits macrophage chemotaxis and activation to attenuate liver ischemia/reperfusion injury via suppressing glycolysis-mediated HMGB1 lactylation and secretion of hepatocytes
Source: Theranostics. 2023 Jul 3;13(11):3856–71. doi: 10.7150/thno.82607 (PMC10334822; doi:10.7150/thno.82607)
Supplement: Supplementary file 1 — Supplementary figures. [file thnov13p3856s1.pdf]

Supplemental Materials

Figure legends

Figure S1. Optimizing experimental conditions for liver ischemia/reperfusion (LI/R) in mouse and hypoxia/reoxygenation (H/R) in hepatocytes.

A. Mice experiments. After ischemia for 1 h followed by reperfusion for the indicated durations, ALT and AST activities in serum of mice were examined. Data are mean ± SD, ** $P < 0.01$ vs. sham mice by one-way ANOVA followed by Tukey's test. n = 6/group for sham, n = 3/group for 3 h reperfusion, and n = 7/group for 6 h reperfusion.

B. Hepatocyte experiments. After hypoxia for 3 h followed by reoxygenation for the indicated durations, ALT and AST activities in culture medium of hepatocytes were examined. Data are mean ± SD, ** $P < 0.01$ vs. normoxia controls by one-way ANOVA followed by Tukey's test. n = 6/group.

Figure S2. Creation of knockin mice with hepatocyte-specific overexpression of HSPA12A (h-Ki).

A. Scheme for constructing conditional *Hspa12a* knockin mice.

B. Cross breeding strategy for generation hepatocyte-specific *Hspa12a* knockin mice using Alb-Cre transgenic mice.

C. Genotyping of offsprings from cross breeding using PCR.

Figure S3. HSPA12A was specifically overexpressed in hepatocytes of h-Ki mice.

A. HSPA12A expression was examined in different organs using immunoblotting. Note that

HSPA12A was only overexpressed in h-Ki livers. * Highlighted the high expression of HSPA12A in liver. n = 5/group.

B. HSPA12A expression was examined in primary hepatocytes and macrophages that isolated from mice. Note that HSPA12A was overexpressed in hepatocytes but not in hepatic macrophages of h-Ki mice. n = 5/group.

Figure S4. HSPA12A knock-in itself did not cause liver injury in mice

A. Serum ALT and AST activities. Serum was collected from WT and h-Ki mice in sham groups for measuring ALT and AST activities. n = 6/group.

B. Oil Red O staining. WT and h-Ki mice frozen liver sections were stained using **Oil Red O** to indicate lipid deposition. Scale bar = 100 μm. n = 4/group.

Figure S5. KO mice aggravate LI/R injury.

A. Serum ALT and AST activities. Serum was collected from mice after sham or LI/R for measuring ALT and AST activities. Data are mean ± SD,** P < 0.01 by two-way ANOVA followed by Tukey's test. n = 6/groups.

B. Histological examination. After sham or LI/R, liver tissues were collected, paraffin-embedded sectioned, and HE stained. Suzuki's scoring was performed and hepatic necrosis areas were measured to indicate histological injury to indicate histological injury. Scale bar = 200 μm. Data are mean ± SD, * P < 0.05, ** P < 0.01 by two-way ANOVA followed by Tukey's test. n = 6/group.

C. Mouse survival. Mice mortality was recorded after LI/R. *P < 0.05 by log-rank test, n = 24/group.

Figure S6. Hepatocyte HSPA12A paracrinally inhibited macrophage activation.

A. **Experimental protocol.** Primary hepatocytes were exposed to H/R for 6 h/3 h, and the culture medium was collected as conditioned medium (CM). The hepatocyte CM was then added to Raw264.7 macrophages.

B. **Macrophage morphology.** After incubation with hepatocyte CM for 24 h, morphology of Raw264.7 macrophages were examined using phase-contrast microscope. n = 5/group.

C. **Gene expression in macrophages.** After incubation with hepatocyte CM for 24 h, Raw264.7 macrophages were collected for measuring expression of the indicated genes using PCR. Data are mean ± SD, ** $P < 0.01$ by Student's two-tailed unpaired *t* test. n = 6/group.

Figure S7. HSPA12A protected hepatocytes against H/R challenge with or without macrophage coculture.

A. **H/R-induced hepatocyte injury.** Primary hepatocytes were exposed to H/R for 6 h/3 h. Culture medium was collected for measing ALT and AST activities. n = 6/group.

B. **Effects of macrophage coculture on the injury of H/R-exposed hepatocytes.** Primary hepatocytes that growing on lower layer of Transwell plate were exposed to H/R for 6 h/3 h and followed by coculture with macrophages that growing on insert membrane. After coculturing for 24 h, medium ALT and AST activities were measured. n = 6/group.

Data are mean ± SD, ** $P < 0.01$ by two-way ANOVA followed by Tukey's test.

Figure S8. Serum HMGB1 level. Serum was collected from mice after sham or LI/R for measuring HMGB1 by ELISA. Data are mean ± SD. ** $P < 0.01$ by two-way ANOVA followed by Tukey's test. n = 8 for sham groups and n = 12 for LI/R groups.

Figure S9. Medium HMGB1 examination.

A. Experimental protocol. Primary hepatocytes were exposed to H/R for 6 h/3 h.

B. Medium of hepatocyte culture were collected for HMGB1 examination by immunoblotting.

Figure S10. HMGB1 was knockdown in primary hepatocytes.

A. Primary hepatocytes were transfected with Hmgb1-siRNA to knockdown HMGB1 expression. The hepatocytes transfected with scrambled RNA served as controls. The efficiency of HMGB1 knockdown was evaluated by immunoblotting. Data are mean ± SD, ** P < 0.01 by Student's two-tailed unpaired t test, n = 6/group.

B. ALT and AST measurement. After primary hepatocytes were transfected with *Hmgb1*-siRNA (Si-*Hmgb1*) and scramble RNA treatment served control, culture medium was collected after 24 hours for measuring ALT and AST activities. n = 6/group.

Figure S11. HMGB1 knockdown reversed the enhancement of H/R injury in HSPA12A knockout hepatocytes.

A. Experimental protocol. Primary hepatocytes were transfected with *Hmgb1*-siRNA to knockdown HMGB1 expression, and hepatocytes transfected with scrambled RNA served as controls. Twenty-four hours later, hepatocytes were exposed to H/R for 6 h/3 h.

B. **ALT and AST measurement.** After H/R experiments, culture medium was collected for measuring ALT and AST activities. Data are mean ± SD, ** $P < 0.01$ by two-way ANOVA followed by Tukey's test. ns, no significance. n = 5/group.

Figure S12. HSPA12A overexpression inhibited the H/R-induced increase of HMGB1 lactylation in hepatocytes.

A. **Klac levels in HMGB1 immunoprecipitates.** After exposed to H/R or normoxia, primary hepatocytes were prepared for anti-HMGB1 immunoprecipitation, and the immunoprecipitates were immunoblotted for lysine lactylation (Klac) and HMGB1. Data are mean ± SD, ** $P < 0.01$ by two-way ANOVA followed by Tukey's test, n = 3/group.
B. **Colocalization of Klac and HMGB1.** After H/R experiments, primary hepatocytes were immunestained with Klac and HMGB1. DAPI was used to counter stain nuclei. Note that the H/R-increased Klac-HMGB1 colocalization was attenuated in cytoplasm of HSPA12A Ki hepatocytes. n = 3/group.

Figure S13. HSPA12A overexpression prevented the H/R-induced increase of glycolysis-derived lactate levels of hepatocytes.

A. **Experimental protocol.** Primary hepatocytes were exposed to H/R for 6 h/3 h.
B. **Extracellular lactate levels.** Lactate levels were examined in culture medium. n = 6/group.
C. **Expression of glycolysis-related genes.** After H/R, primary hepatocytes were collected for examining the indicated gene expression using immunoblotting analysis. n = 4/group.

Data are mean ± SD, ** $P < 0.01$ by two-way ANOVA followed by Tukey's test.

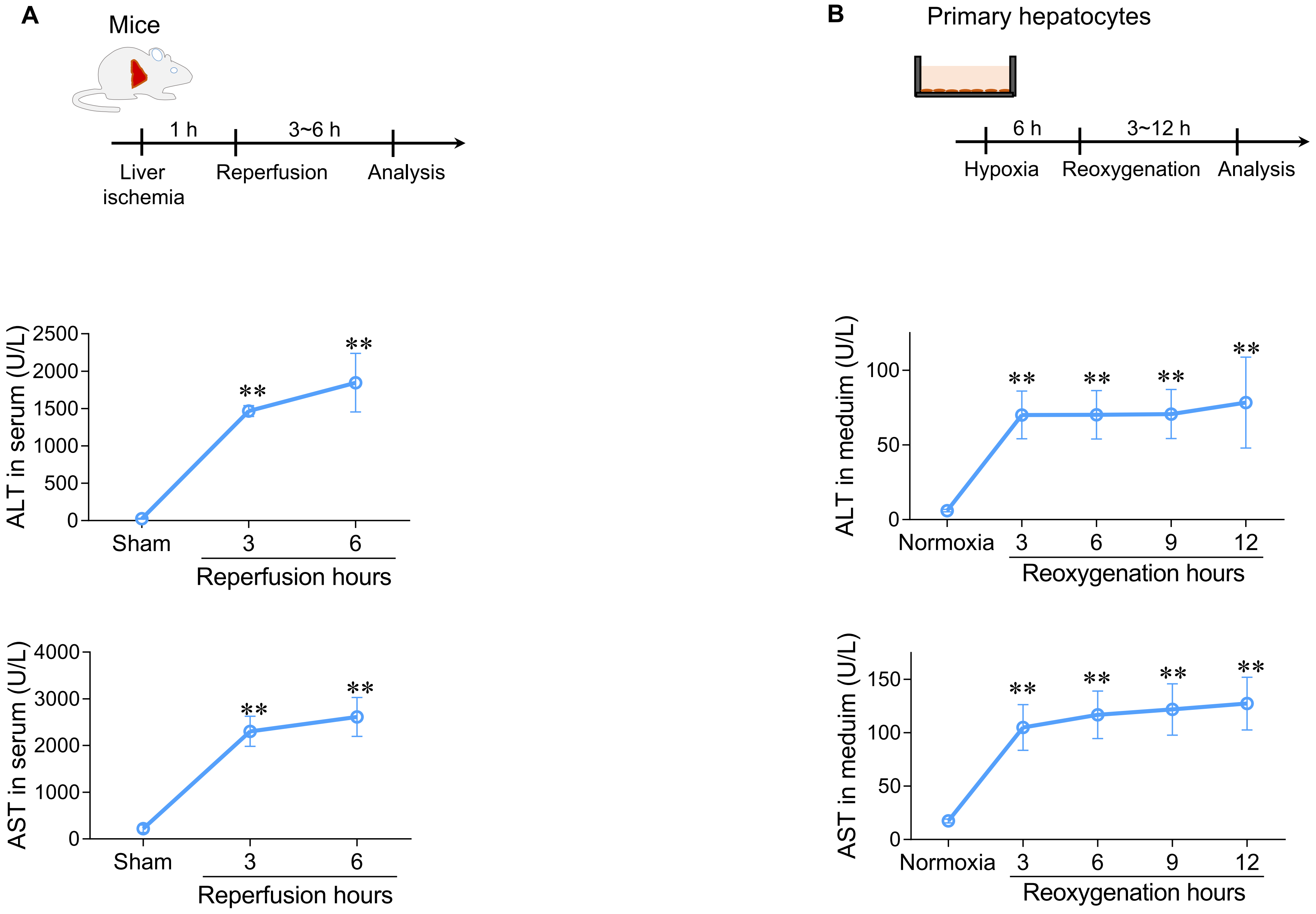
A
Mice
1 h
3~6 h
Liver ischemia
Reperfusion
Analysis
ALT in serum (U/L)
2500
2000
1500
1000
500
0
**
**
Sham
3
6
Reperfusion hours
AST in serum (U/L)
4000
3000
2000
1000
0
**
**
Sham
3
6
Reperfusion hours
B
Primary hepatocytes
6 h
3~12 h
Hypoxia
Reoxygenation
Analysis
ALT in meduim (U/L)
100
50
0
**
**
**
**
Normoxia
3
6
9
12
Reoxygenation hours
AST in meduim (U/L)
150
100
50
0
**
**
**
**
Normoxia
3
6
9
12
Reoxygenation hours

Figure S1

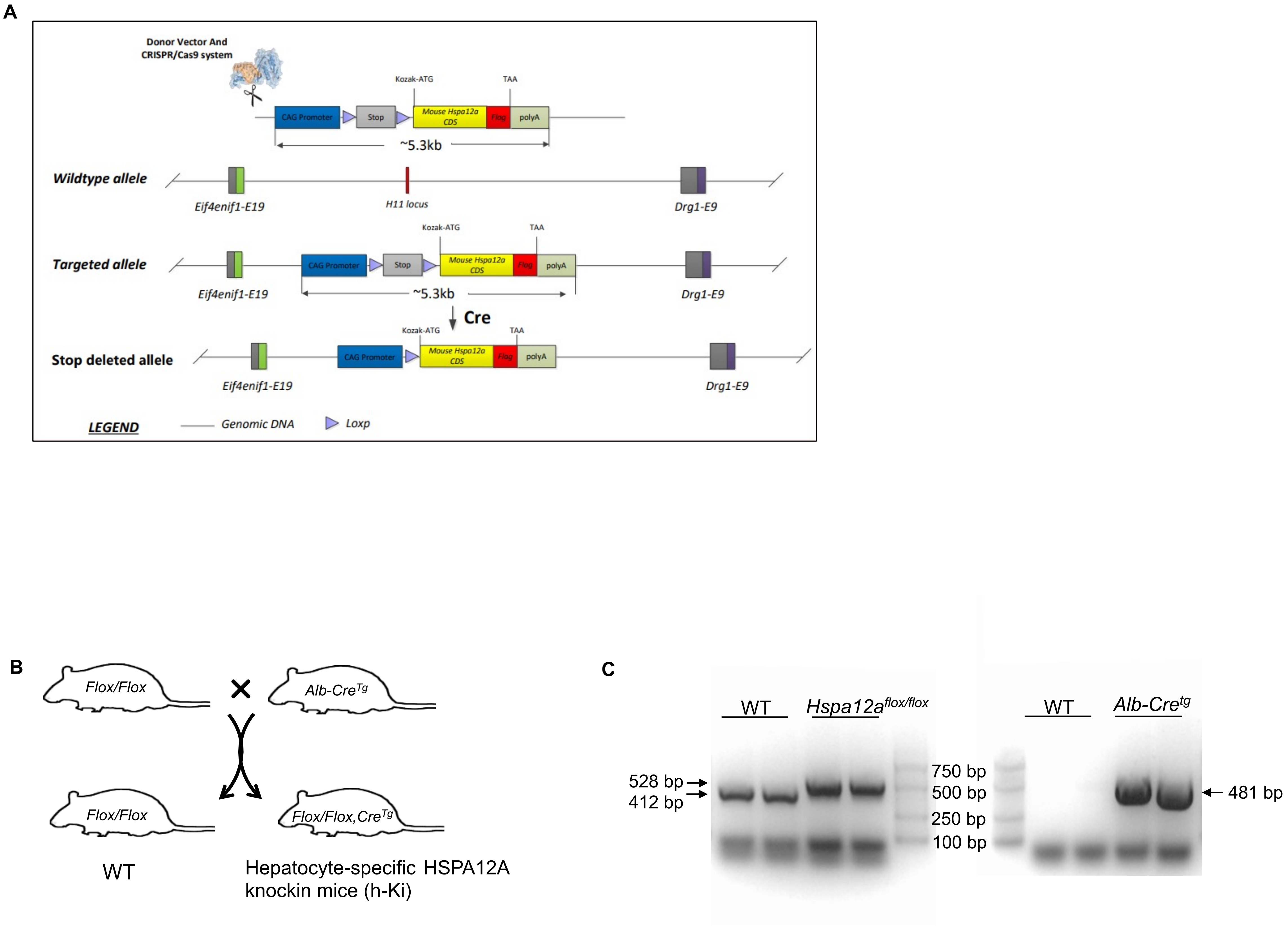
A
Donor Vector And CRISPR/Cas9 system
Kozak-ATG
TAA
CAG Promoter
Stop
Mouse Hspa12a CDS
Flag
polyA
~5.3kb
Wildtype allele
Eif4enif1-E19
H11 locus
Drg1-E9
Targeted allele
Cre
Stop deleted allele
LEGEND
Genomic DNA
Loxp
B
Flox/Flox
Alb-CreTg
Flox/Flox,CreTg
WT
Hepatocyte-specific HSPA12A knockin mice (h-Ki)
C
WT
Hspa12aflox/flox
Alb-Cretg
528 bp
412 bp
750 bp
500 bp
250 bp
100 bp
481 bp

Figure S2

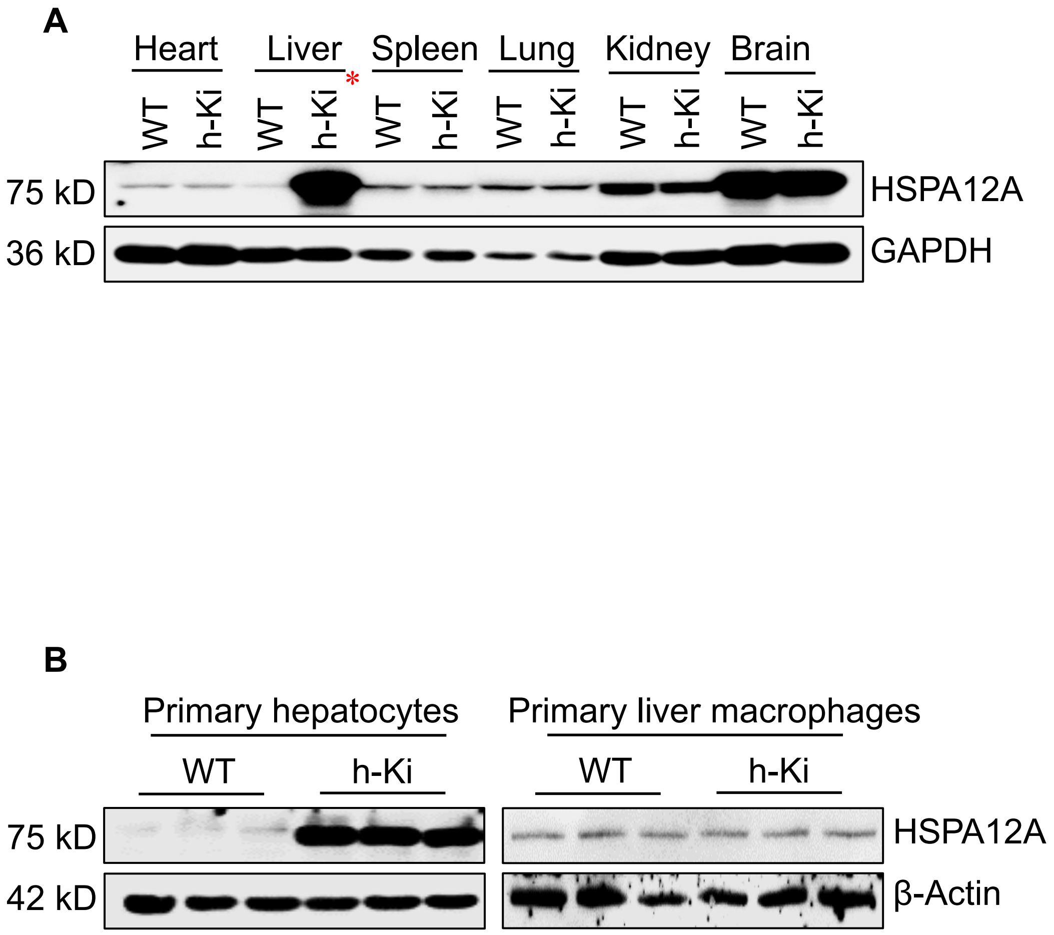
A
Heart
Liver
*
Spleen
Lung
Kidney
Brain
WT
h-Ki
WT
h-Ki
WT
h-Ki
WT
h-Ki
WT
h-Ki
WT
h-Ki
75 kD
HSPA12A
36 kD
GAPDH
B
Primary hepatocytes
Primary liver macrophages
WT
h-Ki
WT
h-Ki
75 kD
HSPA12A
42 kD
β-Actin

Figure S3

A

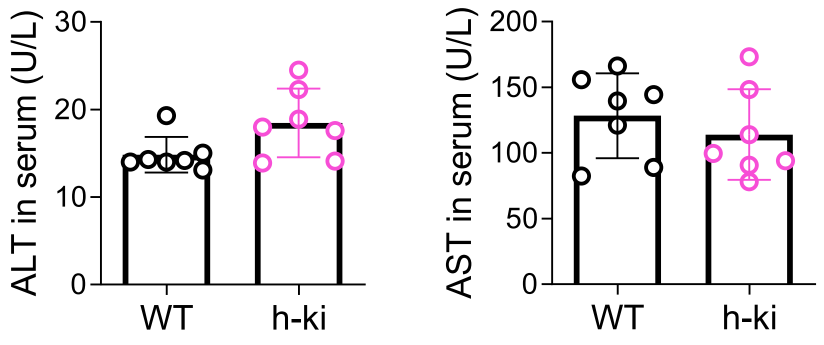

ALT in serum (U/L)
30
20
10
0
WT
h-ki
AST in serum (U/L)
200
150
100
50
0
WT
h-ki

B

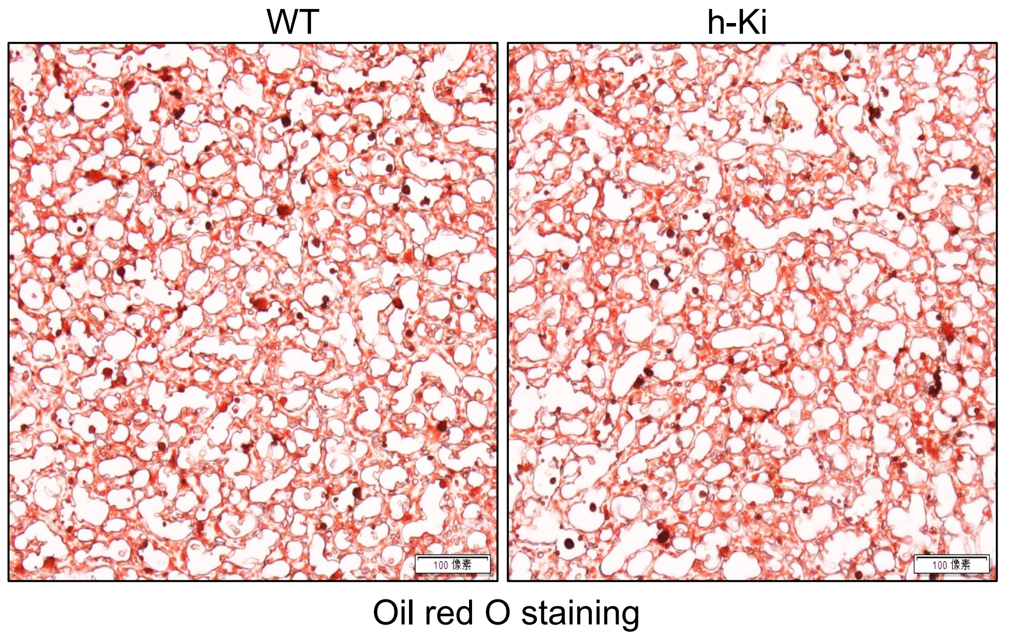

WT
h-Ki
Oil red O staining

Figure S4

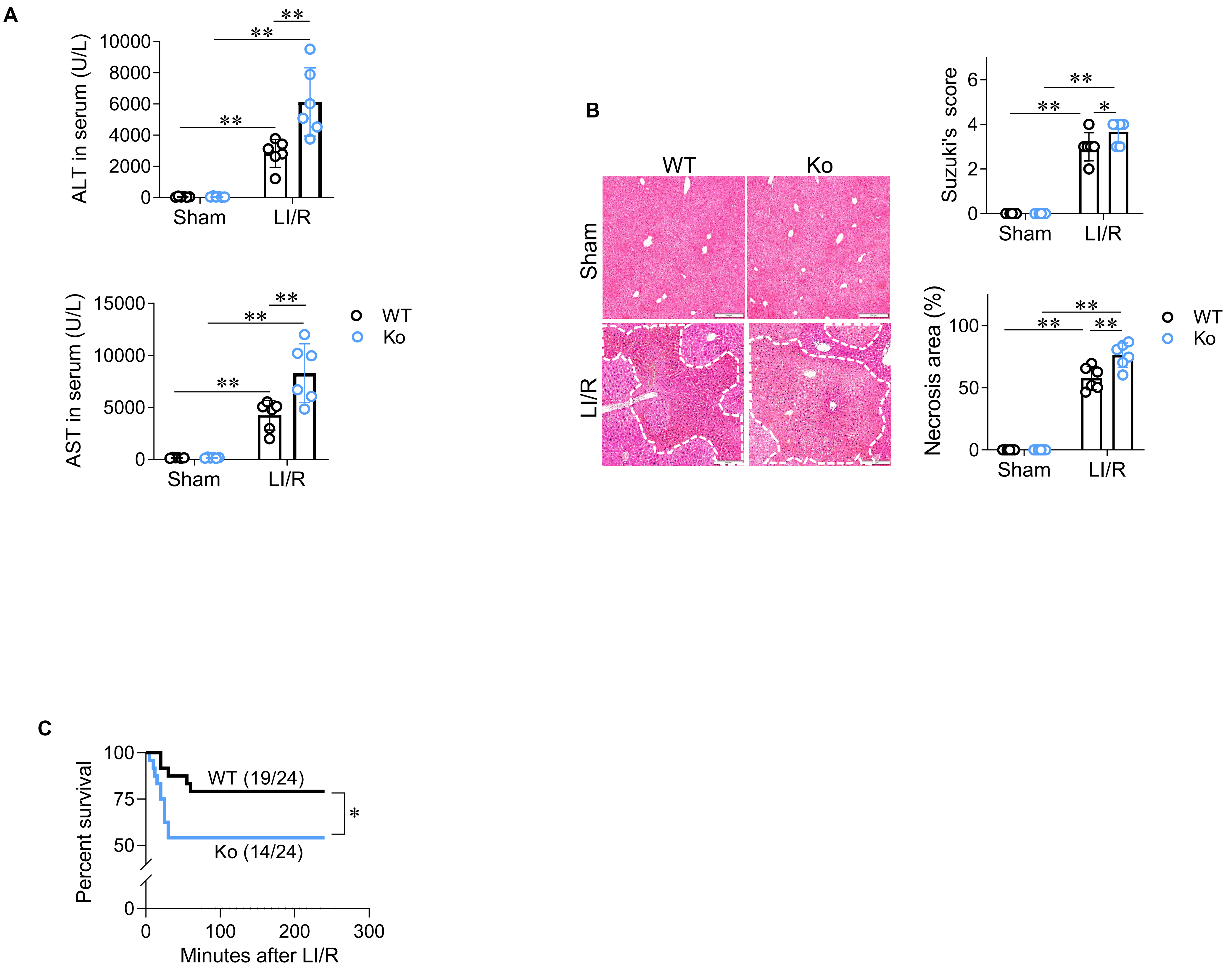
A
ALT in serum (U/L)
10000
8000
6000
4000
2000
0
Sham
LI/R
**
AST in serum (U/L)
15000
10000
5000
WT
Ko
B
WT
Ko
Sham
LI/R
Suzuki's score
6
4
2
*
Necrosis area (%)
100
50
C
Percent survival
100
75
50
0
WT (19/24)
Ko (14/24)
100
200
300
Minutes after LI/R

Figure S5

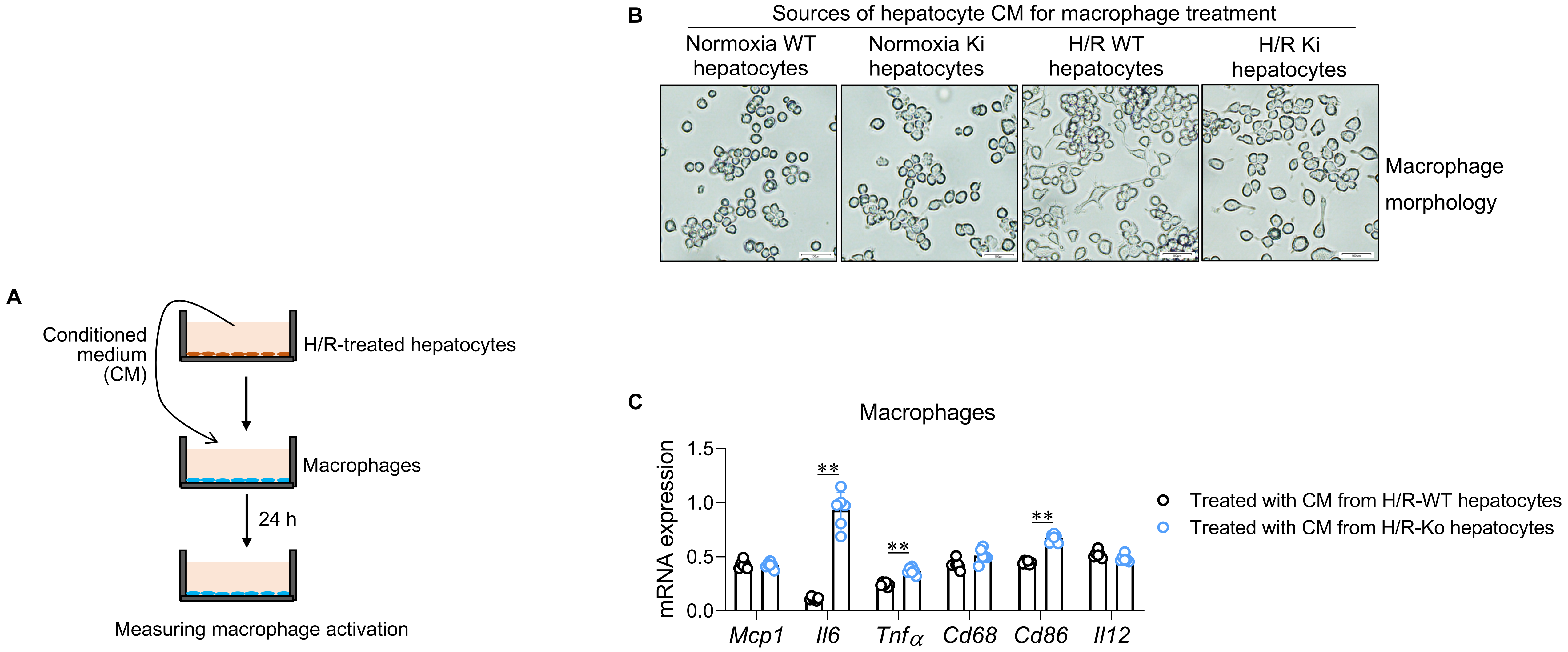
B
Sources of hepatocyte CM for macrophage treatment
Normoxia WT hepatocytes
Normoxia Ki hepatocytes
H/R WT hepatocytes
H/R Ki hepatocytes
Macrophage morphology
A
Conditioned medium (CM)
H/R-treated hepatocytes
Macrophages
24 h
Measuring macrophage activation
C
Macrophages
mRNA expression
1.5
1.0
0.5
0.0
**
**
**
Mcp1
Il6
Tnfα
Cd68
Cd86
Il12
Treated with CM from H/R-WT hepatocytes
Treated with CM from H/R-Ko hepatocytes

Figure S6

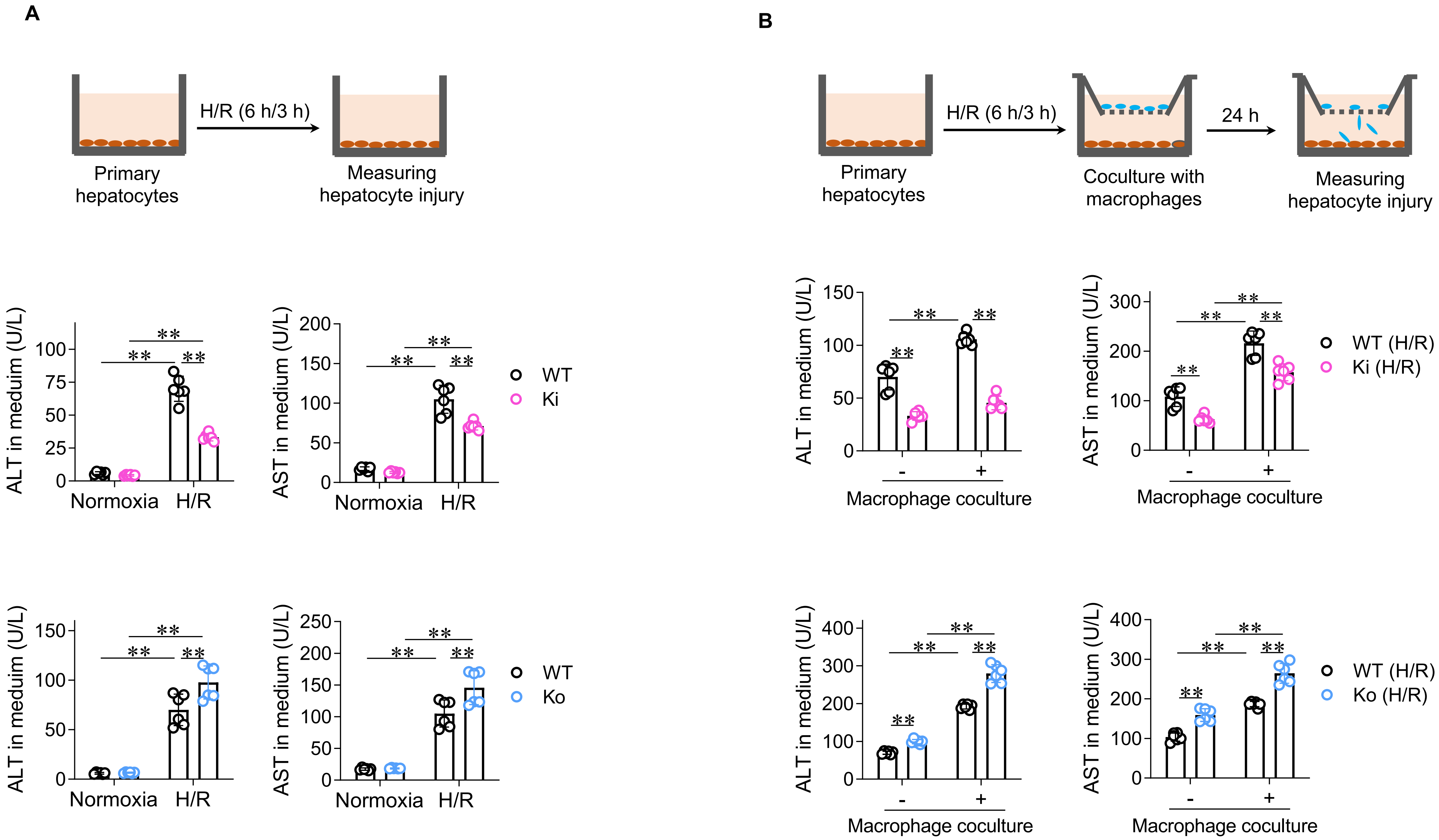
A
Primary hepatocytes
H/R (6 h/3 h)
Measuring hepatocyte injury
ALT in meduim (U/L)
AST in medium (U/L)
Normoxia
H/R
WT
Ki
Ko
**
B
Primary hepatocytes
H/R (6 h/3 h)
Coculture with macrophages
24 h
Measuring hepatocyte injury
ALT in medium (U/L)
AST in medium (U/L)
Macrophage coculture
-
+
WT (H/R)
Ki (H/R)
Ko (H/R)

Figure S7

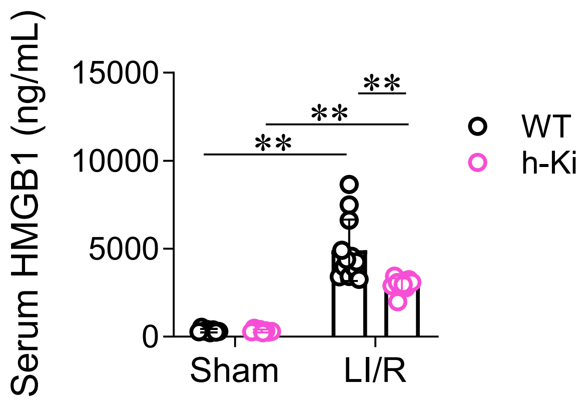

Figure S8

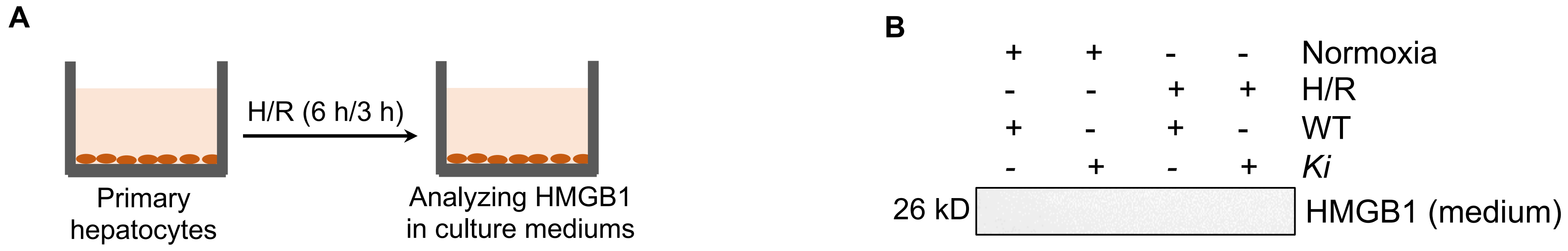
A
H/R (6 h/3 h)
Primary
hepatocytes
Analyzing HMGB1
in culture mediums
B
+ + - - Normoxia
- - + + H/R
+ - + - WT
- + - + Ki
26 kD
HMGB1 (medium)

Figure S9

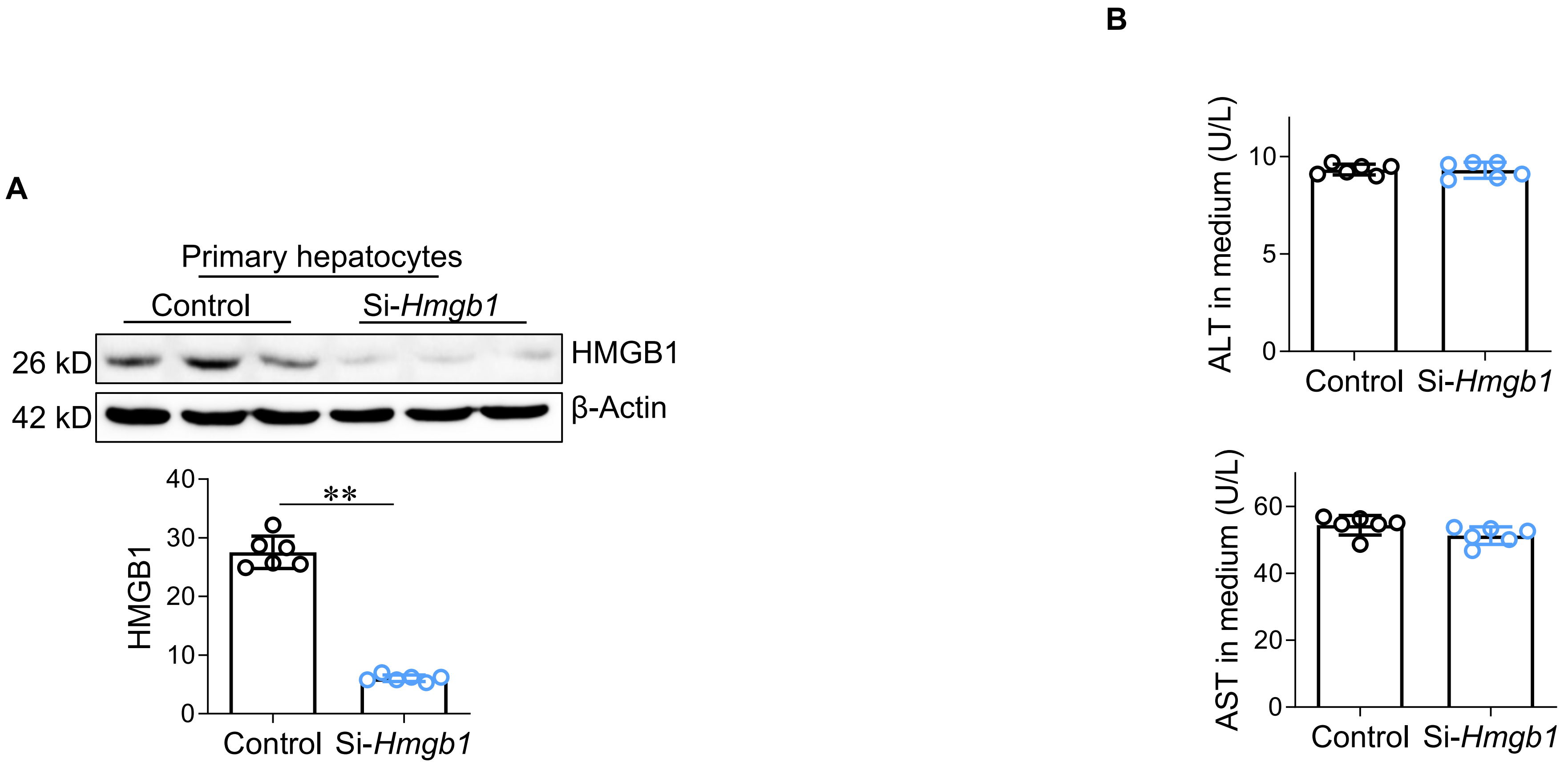
A
Primary hepatocytes
Control
Si-Hmgb1
26 kD
HMGB1
42 kD
β-Actin
HMGB1
40
30
20
10
0
**
Control
Si-Hmgb1
B
ALT in medium (U/L)
10
5
0
Control
Si-Hmgb1
AST in medium (U/L)
60
40
20
0
Control
Si-Hmgb1

Figure S10

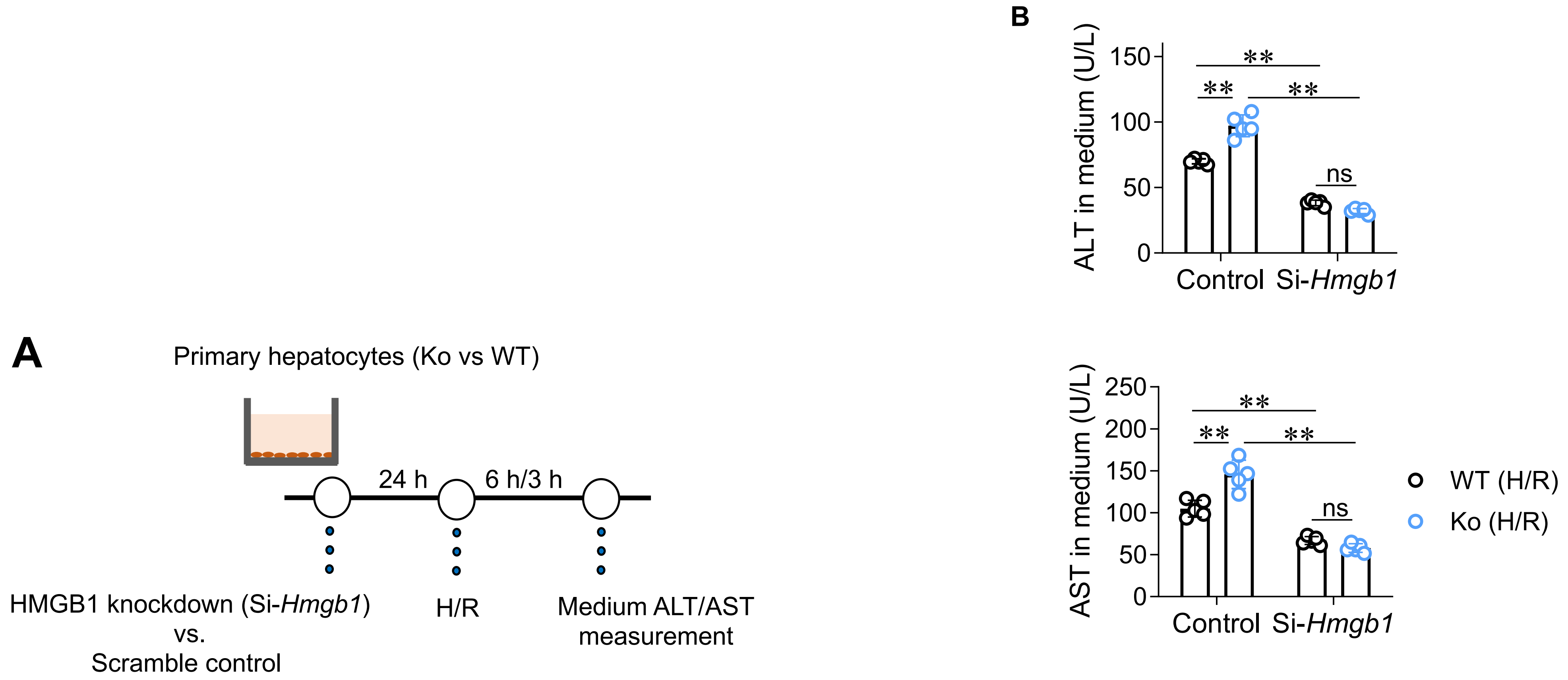
A
Primary hepatocytes (Ko vs WT)
24 h
6 h/3 h
HMGB1 knockdown (Si-*Hmgb1*)
vs.
Scramble control
H/R
Medium ALT/AST
measurement
B
ALT in medium (U/L)
150
100
50
0
**
**
**
ns
Control
Si-*Hmgb1*
AST in medium (U/L)
250
200
150
100
50
0
**
**
**
ns
Control
Si-*Hmgb1*
WT (H/R)
Ko (H/R)

Figure S11

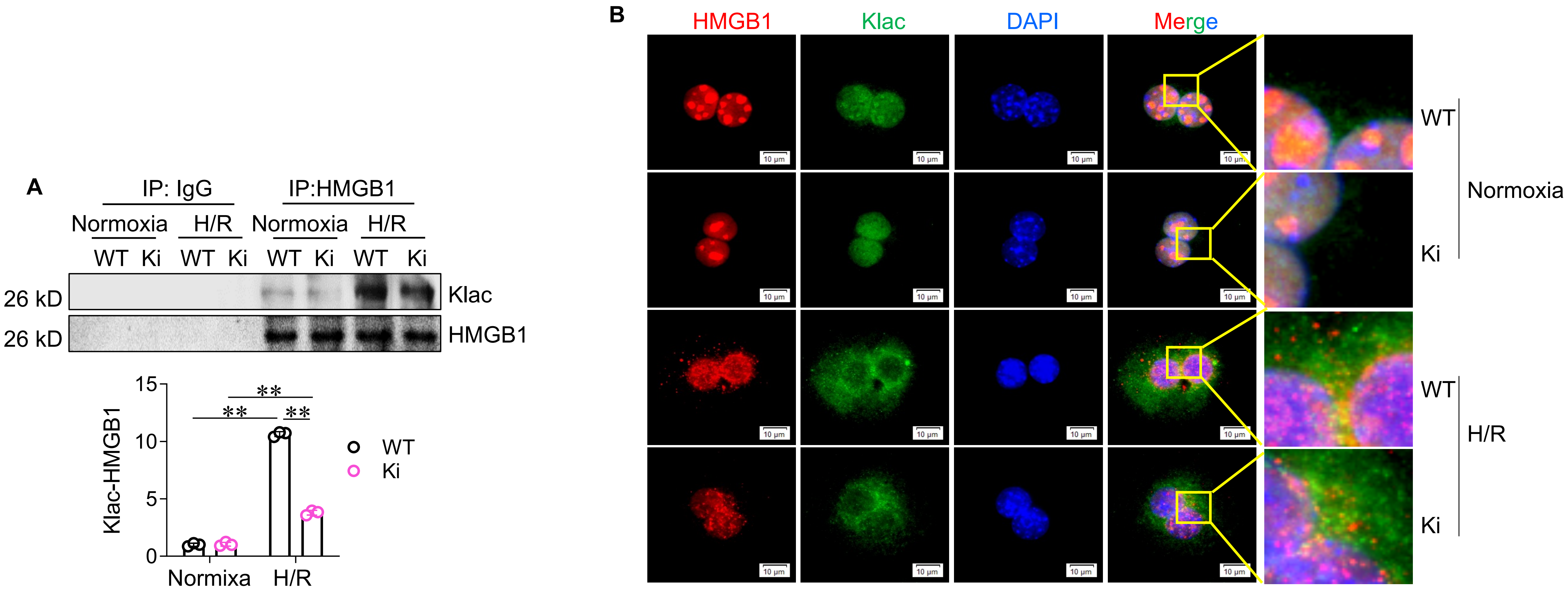
A
IP: IgG
IP:HMGB1
Normoxia
H/R
Normoxia
H/R
WT
Ki
WT
Ki
WT
Ki
WT
Ki
26 kD
Klac
26 kD
HMGB1
Klac-HMGB1
15
10
5
0
**
**
**
WT
Ki
Normixa
H/R
B
HMGB1
Klac
DAPI
Merge
WT
Ki
Normoxia
WT
Ki
H/R

Figure S12

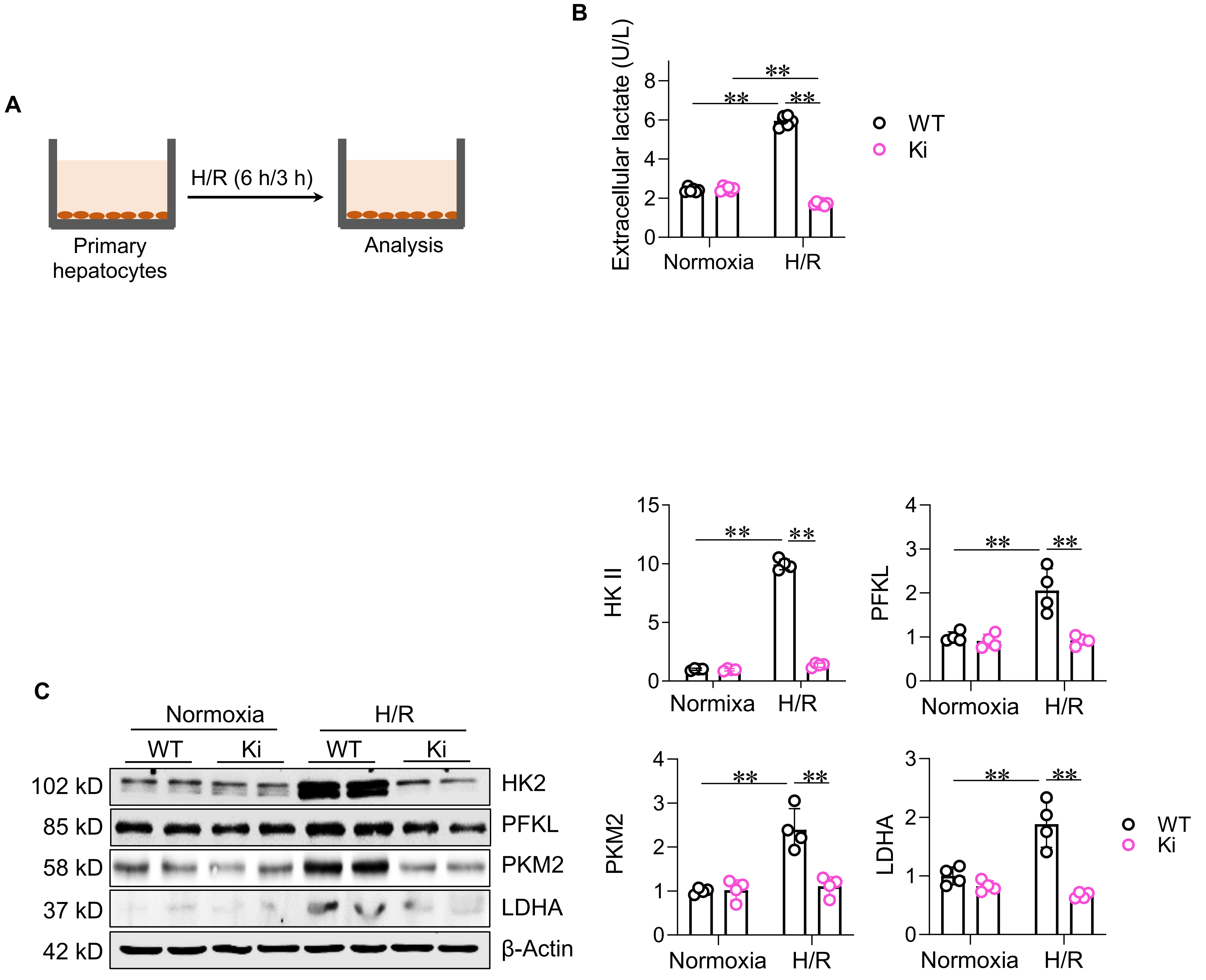

A
H/R (6 h/3 h)
Primary hepatocytes
Analysis
B
Extracellular lactate (U/L)
0
2
4
6
8
**
**
**
Normoxia
H/R
WT
Ki
C
Normoxia
H/R
WT
Ki
WT
Ki
102 kD
85 kD
58 kD
37 kD
42 kD
HK2
PFKL
PKM2
LDHA
β-Actin
HK II
0
5
10
15
Normixa
H/R
PFKL
0
1
2
3
4
Normoxia
H/R
PKM2
Normoxia
H/R
LDHA
Normoxia
H/R
WT
Ki

Figure S13
